# A generative AI-driven interactive listening assessment task

**DOI:** 10.3389/frai.2024.1474019

**Published:** 2024-11-04

**Authors:** Andrew Runge, Yigal Attali, Geoffrey T. LaFlair, Yena Park, Jacqueline Church

**Affiliations:** Duolingo, Pittsburgh, PA, United States

**Keywords:** automatic item generation, listening assessment, interactional competence, generative AI, psychometrics, interactive listening, Duolingo English test

## Abstract

**Introduction:**

Assessments of interactional competence have traditionally been limited in large-scale language assessments. The listening portion suffers from construct underrepresentation, whereas the speaking portion suffers from limited task formats such as in-person interviews or role plays. Human-delivered tasks are challenging to administer at large scales, while automated assessments are typically very narrow in their assessment of the construct because they have carried over the limitations of traditional paper-based tasks to digital formats. However, computer-based assessments do allow for more interactive, automatically administered tasks, but come with increased complexity in task creation. Large language models present new opportunities for enhanced automated item generation (AIG) processes that can create complex content types and tasks at scale that support richer assessments.

**Methods:**

This paper describes the use of such methods to generate content at scale for an interactive listening measure of interactional competence for the Duolingo English Test (DET), a large-scale, high-stakes test of English proficiency. The Interactive Listening task assesses test takers’ ability to participate in a full conversation, resulting in a more authentic assessment of interactive listening ability than prior automated assessments by positing comprehension and interaction as purposes of listening.

**Results and discussion:**

The results of a pilot of 713 tasks with hundreds of responses per task, along with the results of human review, demonstrate the feasibility of a human-in-the-loop, generative AI-driven approach for automatic creation of complex educational assessments at scale.

## Introduction

Listening comprehension is a critical part of language proficiency (Wagner, 2014). Assessment of listening comprehension, however, has long underrepresented the interactional and communicative abilities of the listening test-takers (Aryadoust and Luo, 2023). Large-scale assessments of L2 academic English proficiency ask test takers to take a passive role in comprehending a speaker in a traditional lecture. To tap into the communicative aspect of listening ability, a listening assessment would at most include comprehension questions about a conversation that test takers passively listen to, or have test takers complete a single turn in a conversation (Buck, 2001; Papageorgiou et al., 2021). Aryadoust and Luo (2023) call for a shift in focus in listening assessment to technology-driven constructs in virtual settings such as interacting with others in real-time. To that end, we present a novel assessment of listening comprehension, the Interactive Listening task, that asks test takers to participate and sustain a virtual conversation. We apply recent advances in generative AI (Brown et al., 2020; OpenAI et al., 2024) to the task of automated item generation (AIG, Attali et al., 2022) to generate the conversational content and items used for this task.

The rest of this paper is organized as follows. We first review the current state of automatic item generation and assessments of listening and interactional competence that motivated our work. Next, we present an overview of our Interactive Listening task and describe decisions we made with regards to how we designed the task to assess communicative listening ability. We describe the generative AI-based item generation processes we developed to create a large bank of diverse conversations to use for the task, along with our methods for generating, evaluating and selecting distractors for multiple-choice items. We describe a series of small-scale pilot experiments and their key results that informed task design and administration decisions. Finally, we present the results from a large-scale pilot experiment using 713 Interactive Listening tasks administered as part of a practice test on the Duolingo English Test. We report on feedback from human reviewers for the piloted tasks that provides insights into the quality of the AIG processes, while test taker pilot response data allows us to evaluate the psychometric properties of the tasks.

## Background

### Automatic item generation

The adoption of technology by the field of assessment has moved past a shift in the mode of delivery: from paper-based to computer-based (or internet-based). The current state of technology in assessment can be better described as leveraging technology across the test development, administration, and scoring continuum to improve the ways in which latent traits are assessed. Now, internet-based computerized assessments are making use of advances in technology for a variety of purposes, including developing innovative item types and formats (Sireci and Zenisky, 2006), measuring more complex knowledge, skills, and competencies (Bartram and Hambleton, 2005), implementing automated scoring with immediate feedback to students (Attali and Powers, 2010), offering adaptive, on-demand testing (van der Linden and Glas, 2010), and offering personalized assessments (Suárez-Álvarez et al., 2023). These adoptions have led to an increase in the volume and offerings of assessments, necessitating the need for a significantly larger item bank to accommodate this increased demand (Downing and Haladyna, 2006; Sayin and Gierl, 2024).

Automatic item generation (AIG) may help address the challenge of developing items at a much larger scale than was needed by traditional paper-based assessments (Circi et al., 2023; Gierl and Haladyna, 2012; Irvine and Kyllonen, 2002). AIG in its nascent form has traditionally been implemented using an item model approach whereby a template for a question with parameters is automatically populated with specific values using a computer-based algorithm (Bejar, 2002). For example, the model X + Y =?, where X and Y can be any whole numbers in the range 0–9, has two parameters X and Y. X and Y in the item model can be populated with any single-digit numbers to display the item. A more complex example is “How many pieces of [fruit] will you have if you cut [5] whole [fruits] into [thirds]?” (Attali, 2018), where the text in parentheses represent parameters (numeric or text). This type of traditional AIG has successfully been used to create items in diverse content areas, such as mathematics word problems and medical diagnosis questions (Haladyna, 2013), expanding the potential number of items with set item models facilitating a construct-driven approach to item development (Embretson and Yang, 2006; Whitely, 1983).

While being fairly useful in content areas where item models are easier to specify, the traditional item model approach to AIG comes with its limitations (von Davier, 2018). One is that it is not easily applicable to other content areas: for instance, second language (L2) reading proficiency, where a template would have to be constructed for each question for each passage (Bolender et al., 2023). Another is that the item model approach relies on highly skilled content experts to create the models and therefore can be costly (Kosh et al., 2018). Due to these shortcomings, the use of AIG as a technique to generate test content has been limited to relatively simple tasks (Attali et al., 2022).

### Large language models and AIG

An alternative to the traditional item model approach to AIG is a generative AI-based approach leveraging recent advances in large language models (LLMs). Language modeling is capable of generating a large amount of text based on limited input, drawing from a probabilistic model of language. Language models based on neural transformer architectures (Devlin et al., 2019; Radford et al., 2019; Vaswani et al., 2017) were previously limited in their applications to AIG as they required a large amount of expert-annotated training data, computing power, and lengthy model development to update the underlying model to accomplish a particular task. These limitations are addressed by OpenAI’s GPT-3 and its subsequent GPT-4 models (Brown et al., 2020; OpenAI et al., 2024) that can generate novel content based on fewer than 10 examples without the need to update the underlying model, referred to as “few-shot” prompting in the context of LLMs (Brown et al., 2020). Additionally, GPT models can be specified to generate the output in any desired format, such as well-formatted, fully functioning HTML code, or a paragraph with comprehension questions. These advantages allow AIG with GPT-based models to be freed from the notion of an item model and expand into item types that cannot be succinctly captured with templates, all the while without putting significant strains on resources.

Generative AI-based approaches have since been used successfully to create more complex assessments that were difficult to construct with the item model approach, from reading passages (e.g., Attali et al., 2022; Bezirhan and von Davier, 2023) and listening stimuli (Aryadoust et al., 2024) to distractors to vocabulary questions (Zu et al., 2023) and reading/listening comprehension items (Attali et al., 2022; Sayin and Gierl, 2024). Despite the rich potential to create content at scale for innovative tasks, generative AI-based approaches have not yet been leveraged fully in high-stakes testing (an exception is Attali et al., 2022). One such area that generative AI-based approaches may provide a solution for is creating rich contexts for listening that allows for a much more in-depth specification of the construct in the assessment of listening. Our work in particular builds on the techniques described in Attali et al. (2022) by using large language models to generate tens of thousands of conversations for an innovative assessment of interactional competence - something that has been historically difficult to create at scale. While Attali et al.'s (2022) work uses only a handful of the generated passages as potential sources of distractors for any one item, we take advantage of structural similarities across conversations to re-use the full set of dialog lines from all generated conversations as potential distractors for multiple choice tasks. This allows for the development of a robust content bank that can continuously grow and support future rounds of item generation.

### Dialog generation

Dialog generation has primarily been researched as a way of augmenting datasets used for training dialog systems. Initial datasets for these tasks were typically collected using crowd workers, which can be expensive and potentially require significant training for more specialized tasks (Dinan et al., 2019; Liu et al., 2021; Gopalakrishnan et al., 2019; Zhang et al., 2018). Researchers have therefore explored data augmentation techniques to create new dialog examples to supplement these existing datasets (Chen and Yang, 2021; Sun et al., 2023). One line of research for whole-dialog generation uses individual simulators for both users and agents, which are trained on existing datasets to help create new instances. This approach has most commonly been applied to task-oriented dialog generation (Papangelis et al., 2019; Hou et al., 2019), though it has also been applied successfully to open-domain and knowledge-grounded dialog generation (Kim et al., 2023; Mohapatra et al., 2021; Wu et al., 2022).

More recently, several works have explored dialog creation using or assisted by LLMs due to LLMs’ strong few-shot learning capability which allows the creation of new dialogs using a very small handful of examples. While the simulator method could only generate dialogs in the domain of their training sets, LLM-based dialog generation allows for quick development of large datasets in new domains. One such approach that is similar to ours for dialog generation is the concurrently developed SODA dataset (Kim et al., 2023). Their approach uses triples automatically harvested from a commonsense knowledge graph and uses OpenAI’s text-davinci-002 model to first convert them into short narratives and then use those narratives to generate dialogs. Their approach is focused on producing a highly diverse range of social contexts but does not attempt to balance the resulting dataset for any specific conversational characteristics. Another example is the PLACES dataset (Chen et al., 2023) that combined a small sample of hand-crafted example conversations with topics extracted from the Feedback for Interactive Talk & Search Dataset (Xu et al., 2023) to generate new dialogs. Additionally, Lee et al. (2022) created the PERSONACHATGEN dataset to extend the PERSONACHAT dataset (Zhang et al., 2018) with synthetic dialogs using two GPT-3 instances as separate agents, each seeded with a synthetically generated persona. Other works have used partial conversations from existing datasets as the input seeds and used pre-trained language models (Chen et al., 2022) or language models fine-tuned on separate datasets (Zheng et al., 2023) to create new instances by completing the partial conversations. These works, while useful, do not envision the generated dialogs to be used as content for language assessment but primarily as training data for dialog systems, facilitating the need for a separate method for dialog creation that is tailored to standardized large-scale proficiency assessment in order to ensure construct alignment of the generated conversations.

### Assessment of listening

The construct of listening has been defined and operationalized in various ways (see Aryadoust and Luo, 2023) but the specific ways in which the construct of listening has been elicited and measured in large-scale standardized assessments has been fairly limited. Buck (2001) proposes five subskills as a starting definition of the listening construct that work in tandem to arrive at listening comprehension: knowledge of the sound system, understanding local linguistic meanings, understanding full linguistic meanings, understanding inferred meanings, and communicative listening ability. To elicit these skills in a reliable manner, large-scale standardized tests mainly make use of discrete-point comprehension questions that ask test takers to listen to a stimulus, read (or listen) to a question, and then choose the most appropriate answer via a multiple-choice format or a true/false format (Carr, 2011; Park et al., 2022; Wagner, 2014). Other formats include asking test takers to fill in the gaps of summaries or complete a flow chart, a diagram, or a table (Suvorov and Li, 2023).

While these assessment formats are successful at tapping into the five aforementioned subskills, they fall short of extending them to communicative situations where test takers are using the processed aural information to interact with another person, which makes up most real-life listening domains (Wagner, 2014). Addressing this concern via conversational stimuli is limited as test takers remain passive listeners, with tasks to be completed after the conversation has taken place rather than during. By positioning test takers as passive listeners in conversations rather than active participants, the construct of listening is specified in a way that favors one purpose of listening, comprehension, over others such as conversation and interaction (Aryadoust and Luo, 2023). This problem of construct underrepresentation threatens our ability to make accurate inferences about a test taker’s listening proficiency if the purpose of using the assessment is to determine whether they are adequately prepared for situations that require them to make use of their listening abilities in university settings in which they are expected to converse with other people such as engaging in discussions in office hours, participating in service encounters, and communicating with their peers.

Tasks that address the concern of construct underrepresentation by appointing test takers as active participants in conversations may be able to further broaden their construct coverage with the help from generative AI. An example of tasks that situate test takers in conversations would be a discourse-completion task typically employed in assessment of pragmatic competence (Brown, 2018; Buck, 2001), where participants listen to a conversation and complete the last turn in the conversation. An instantiation of this task in a large-scale standardized context can be seen in Papageorgiou et al. (2021) which has test takers listen to a conversation and complete the last turn in the conversation. Extending this type of task beyond having test takers complete the last turn to them fully engaging in the conversation would allow a fuller specification of the listening construct in interactional situations.

### Assessment of interactional competence

Interactional competence is defined as “the ability to co-construct interaction in a purposeful and meaningful way … supported by … aspects of topic management, turn management, interactive listening, breakdown repair and non-verbal or visual behaviours” (Galaczi and Taylor, 2018, p. 224). The ways in which interlocutors exercise their interactional (sub)skills according to the speech events and speech acts, which are in turn based on speech situations, facilitate the characterization of interactional competence as a tree (Galaczi and Taylor, 2018). An example of a speech situation would be office hours between a student and a professor in a university setting. The speech event is the actual conversation that transpires between the two interlocutors, who subsequently employ interactional subskills such as asking questions for topic management and maintaining for turn management.

Assessing interactional competence has been a notoriously thorny problem for large-scale standardized high-stakes language proficiency exams (Dai, 2024; Roever and Dai, 2021) due to difficulties with scoring, operationalization of the construct, and finding the right balance between reliability and construct coverage (Galaczi and Taylor, 2018). Typically, measurement of this construct, or a part of it, has been targeted in speaking tasks with interactive task formats such as oral proficiency interviews (OPIs) and paired tasks (Roever and Kasper, 2018; Youn, 2020). These interactive formats provide opportunities to measure interactional competence, but they also introduce possible sources of error as well.

In OPIs, human examiners are paired with test takers for a face-to-face spoken interaction to elicit language samples to rate test takers’ oral proficiency. OPIs have been shown to be distinct from naturally occurring conversation in the TLU domain in terms of turn-taking, topic control, and question-response patterns (Johnson and Tyler, 1998; Seedhouse, 2013; van Lier, 1989; Young and He, 1998). The source of these differences from conversation has been found to be due to differences between the task and TLU domain with respect to the actual purpose for interaction in the TLU domain and the power difference between interlocutors. All of these threaten the generalizability of a test taker’s performance from OPIs alone to how they would actually utilize their interactional competence in the TLU domain (Plough et al., 2018; Staples et al., 2017; van Lier, 1989).

While the interview-based format of OPIs has been found to deviate from core elements of conversation and interactional competence, the format of guided role plays has demonstrated more accurate alignment to interactional elements in conversation (Kormos, 1999). Typically, the two test takers have a common goal, or purpose, for communicating which creates a language test situation that is more aligned with the TLU domain with respect to interactivity (Davis, 2009; Galaczi, 2008). Additionally, the examiner is usually an onlooker or a facilitator, which mitigates against power differential effects that are found in interview formats (Davis, 2009). However, research has shown that characteristics (such as gender, extroversion, and proficiency) of interlocutors in paired tasks may or may not affect the language that a test taker uses and the score that they receive on the task (Davis, 2009; Galaczi and Taylor, 2018; Iwashita, 1996; Nakatsuhara, 2011). Galaczi and Taylor (2018) argue that this interlocutor induced variability should either be conceptualized as construct irrelevant variance and removed from task design and scoring or conceptualized as construct-relevant variance and accounted for in task design and scoring.

One promising direction for automating and scaling the assessment of interactional competence is the use of spoken dialog systems (SDS). These systems use a combination of automated speech recognition (ASR), a dialog management module, and text-to-speech (TTS) to receive spoken responses from a human and respond to them according to the goals defined by the developers of the system. This allows test takers to go through a full conversation with the system in a fully automatable way. SDS have a long history of research and application in computer-assisted language learning (e.g., Eskénazi, 2009; Su et al., 2015; Timpe-Laughlin et al., 2017), but their application to language assessment has been more limited until the last few years (Litman et al., 2018). Recent studies of SDS have laid the groundwork for using these systems to assess interactional competence by establishing that human to computer interactions display pragmatic functions similar to those in human-to-human communication (Dombi et al., 2022) and can be used to assess interactional competence (Ockey et al., 2023). Human-to-computer interaction has also been found to be comparable to human-to-human interaction (Ockey and Chukharev-Hudilainen, 2021), with the potential to improve task design with teacher feedback (Timpe-Laughlin et al., 2020).

SDS-based assessment tasks have some limitations that make them difficult to deploy for large-scale, high-stakes testing. The primary issue is the difficulty of scaling them to produce new assessment tasks. SDS-based assessment tasks typically combine pre-written dialog trees for the system’s responses and a set of rules regarding the content of the user’s responses to govern the transition from one line in the script to the next (Chukharev-Hudilainen and Ockey, 2021; Gokturk and Chukharev, 2024; Karatay, 2022; Timpe-Laughlin et al., 2017). These rules are usually constructed by hand using combinations of keyword and regex matching, with keywords sourced through manual analysis of data produced either by the researchers (Chukharev-Hudilainen and Ockey, 2021) or crowd workers (Ramanarayanan et al., 2016). As a result, each individual task requires a tremendous amount of manual effort to construct, which constrains the range of situations and interaction structures that can be assessed as well as the number of items that can be created. In addition, issues such as the lack of authenticity in interaction, difficulty in being understood by the system, or the system providing non-meaningful responses could negatively impact test taker performance and lead to issues of fairness (Gokturk and Chukharev, 2024), all of which pose threats to the validity of test scores in high-stakes tests.

## The interactive listening task

In this paper we describe the development of a new task which we call the Interactive Listening task. It overcomes the limitations of traditional listening tasks by bringing in test takers as active participants in a conversation. In this task, test takers are in a role-play setting. They listen to their interlocutor and select the best turn to move the conversation forward. They then receive immediate feedback about the correct answer and the process of listening to the next turn, selecting the best response and receiving feedback repeats itself. This multiple-choice format, coupled with generative AI, extends previous tasks assessing listening beyond answering comprehension items and completing the first or last turn of a conversation. The task also controls for interlocutor effects that constrain the interactions in speaking tasks by introducing language variation that is relevant to the TLU domain. From the perspective of speaking tasks, it is an indirect measure of speaking as turns are selected rather than spoken.

### Task overview

**Figure 1:**
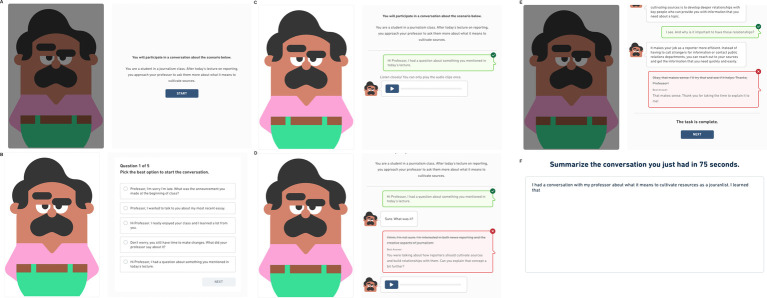
Walkthrough of an interactional competence task. The task starts (A) with a scenario that describes who the test taker is talking with and for what purpose. After they click the start button, the conversation begins. In this task (B), the test taker makes the opening turn of the conversation. When the test taker selects the best answer for the turn (C), they receive feedback through color (green) and a check mark. Test takers receive visual feedback when they select an incorrect option (D) through color (red) and an “x” in the upper right corner of the dialog box. This process repeats until the task is complete (E). Test takers may review the conversation before moving to the summary task (F), where they summarize the content of the conversation.

Figure 1 demonstrates how a test taker would proceed through the Interactive Listening task.

Test takers first read a short description (henceforth referred to as a scenario) which outlines the goal of the conversation, the test taker’s role, and the interlocutor’s role. The interlocutor is represented as an animated character drawn from the cast of the Duolingo World Characters (Chiu et al., 2021; Hartman, 2020). The interlocutor lines are turned into audio stimuli using custom text-to-speech (TTS) models created for each character. Test takers listen to the character speak their lines in the conversation and then select the option that best continues the dialog, taking into account the scenario and prior conversation turns. Each Interactive Listening task consists of 5–6 of these exchanges between the test taker and the character. Finally, test takers produce a written summary of the conversation they participated in. For this paper, we focus our discussion of task and item performance on the multiple-choice items.

Conversations feature topics and roles that are common in university settings. In all conversations, the test taker plays the role of a student, while their interlocutor may be a fellow student or a professor, referred to as student–student and student-professor conversations, respectively. Conversation topics cover a range of real-world communicative purposes, such as asking for and giving advice, making requests, gathering information, and making plans. For example, test takers may need to set up plans to work on a group project with another student, request a letter of recommendation from a professor, or give advice to a friend about what courses to take. The situational context of the task aligns with real-world situations for interacting with peers and professors in university contexts in terms of the topics, the reasons for the conversation, and the relationship between interlocutors.

### Task design considerations

We use generative AI to support the development of the Interactive Listening task for to its ability to scale the production of tasks (and turns within a conversation) to cover the spectrum of possible topics, purposes for communicating, and communicative settings in an extended conversational task. Where traditional discourse completion tasks typically target single turns in a conversation (usually an opening turn or a closing turn), a task that includes measurement of how test takers navigate an entire conversation creates the possibility to assess more aspects of interactional competence within a single task.

The Interactive Listening task uses predetermined non-branching conversation paths rather than dynamically generated conversations based on test-taker responses. Predetermined conversation paths mean that subsequent turns are not reactive to prior turns; rather it is the test taker’s task to identify which option out of a set of presented responses leads toward task completion. The predetermined conversation paths with the multiple-choice format, while somewhat limited in the extent to which the test taker can nominate the topic and the direction of the conversation, provide several advantages, including efficient and construct-aligned item generation and review process, mitigation of risks due to unpredictability, and the opportunity to provide test takers with feedback.

The combination of offline LLM generation and human review allows us to expedite the item generation process while ensuring that the resulting items meet the quality standards necessary for a large-scale high-stakes English proficiency test. The unpredictability of dynamic generation, on the other hand, makes it difficult to maintain consistent quality standards for the items, while at the same time introducing a potential vulnerability through adversarial attacks intended to trigger unexpected behavior from the model, commonly referred to as “jailbreaking” (Wei et al., 2023). Dynamic generation also introduces the risks of model hallucination, in which the model invents new facts or fails to stay consistent with information provided by the model in earlier turns of the dialog (Ji et al., 2023). As the task is centered around academic situations, self-consistency and consistency with real-world information are both critical to avoid potentially confusing test takers. While there has been a large body of work attempting to improve self-consistency (Li et al., 2020; Song et al., 2020) and consistency with external knowledge bases (Rashkin et al., 2021; Shuster et al., 2021; Sun et al., 2023), these solutions are not perfect and introduce significant additional risk and complexity to the task design. Predetermined conversation paths address this by enabling human review of the content that can mitigate the risk of hallucinations and ensure fairness in the generated content (Belzak et al., 2023). Feedback from human reviewers can additionally be used in a human-in-the-loop process to improve the AIG processes and the quality of the generated content. Finally, static conversations allow us to better ensure that the language used in the conversation adheres to aspects of the communicative contexts that we are interested in modeling, such as the power dynamic between the participants.

Additionally, we use a single path through the conversation, rather than modeling each item as a dialog tree because while it would allow test takers more control over the direction of the conversation, it would also introduce significant generation and psychometric modeling issues. Given our decision to not use dynamically generated system responses, a task that supports branching the conversation based on open-ended responses from the user would require a complex and highly specialized workflow for every single individual item created (ex: Timpe-Laughlin et al. (2017) p. 5). As a result, all prior work using branching conversation as a basis for assessment has typically been limited to 2–4 unique items (Evanini et al., 2014; Chukharev-Hudilainen and Ockey, 2021; Karatay, 2022). For a high-stakes test, we must be able to regularly create large numbers of new items to refresh the bank in order to ensure the security of the test, making this limitation infeasible. Another alternative would be to develop branching multiple choice items, but this approach also has significant issues. As the task is administered adaptively based on the test taker’s estimated proficiency, each branch in a given conversation would need to have similar psychometric properties to provide comparable item information across branches at the same turn index. This would be nearly impossible to guarantee during item generation or from a human review process for conversations with branches that require significantly different potential sets of options. While this design could work for weakly ordered conversations that would support shuffling the order of the turns in the conversation, such as asking for information about a list of classes or the types of slot-filling tasks common in task-based dialog systems, it would unacceptably limit the range of interaction types we could assess. This approach would also require significantly more data collection during piloting to support calibration of all potential branches and could result in significant numbers of items being discarded due to poor measurement quality.

From an assessment perspective, the Interactive Listening task is a type of discourse-completion task. However, whereas existing tasks (e.g., Papageorgiou et al., 2021) have participants listen to a conversation and complete the last turn in the conversation (by selecting the best option in a multiple-choice format), our task is an interactive multi-turn discourse-completion task that asks participants to repeatedly select the next turn that best fits the conversation thus far. The interactive element of this task is achieved by providing immediate feedback about participants’ choices. Test takers who select the wrong option for a given turn are shown the correct one before moving to the next step in the conversation. This innovative interactive nature of our task allows test takers to take a much more active role in the conversation in a way that can potentially broaden the task’s construct coverage.

### Content and item generation

**Figure 2:**
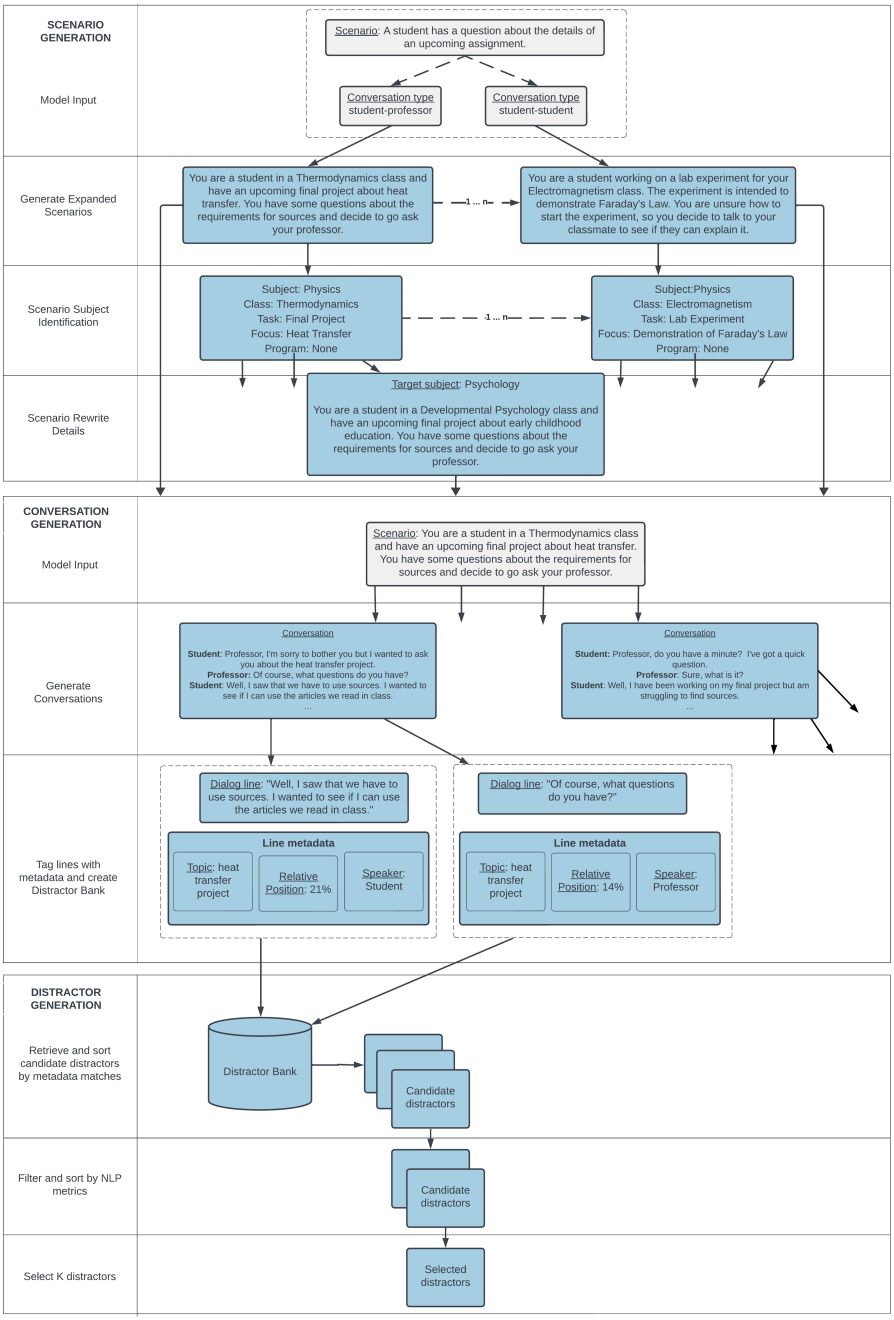
Example of a base scenario and an expanded version with additional details.

This section outlines the processes used to create the conversations and options required for the Interactive Listening task. We used the GPT-3^1^ (Brown et al., 2020) family of LLMs throughout the development of this task, specifically the text-davinci–002^2^ model. Figure 2 shows the workflow we used to create the conversations and options, systematically progressing from the initial generation of conversational scenarios to the formulation of conversations and then finally selection of distractors.

### Conversational scenarios

Our approach to conversation generation is similar to the concurrently developed method used by Kim et al. to create the SODA dataset (2023). While their approach uses automatically extracted triples to seed the generation of short narratives (or conversational scenarios), we use a list of 130 short descriptions of a broad range of typical conversational purposes between two students or between a student and a professor in academic contexts. These scenarios were selected by assessment experts to cover a wide range of basic interactive tasks such as requesting help, asking for advice, providing feedback or recommendations, seeking information, discussing and comparing options, and describing a recent experience (Council of Europe, 2020). We will refer to these as the base scenarios.

For each of these base scenarios, we used a few-shot prompting approach (Brown et al., 2020) with GPT-3 to expand these scenarios with additional details such as specific classes or academic subjects, the type of assignment, the feelings and preferences of the participants, and the relationship between the participants. These additional details in turn can then be used by the model when generating conversations to produce more distinctive and varied conversations from a single hand-crafted input.

For each of our base scenarios, we created 50 detailed scenarios using GPT-3. We generated them five at a time for each base scenario, providing 3–5 examples as input (see Appendix A) that were randomly selected and shuffled from a group of 10 examples to increase the diversity of the outputs. Even with a high temperature value that encourages more diverse output, this approach produced a significant number of near-matching scenario descriptions. We used the sentence-transformers library^3^ (Reimers and Gurevych, 2019) to embed them into a vector space and calculated pairwise cosine similarity between all generated scenarios, removing those with very high cosine similarity to a previously generated scenario. This resulted in 3,900 detailed scenarios.

**Figure 3:**
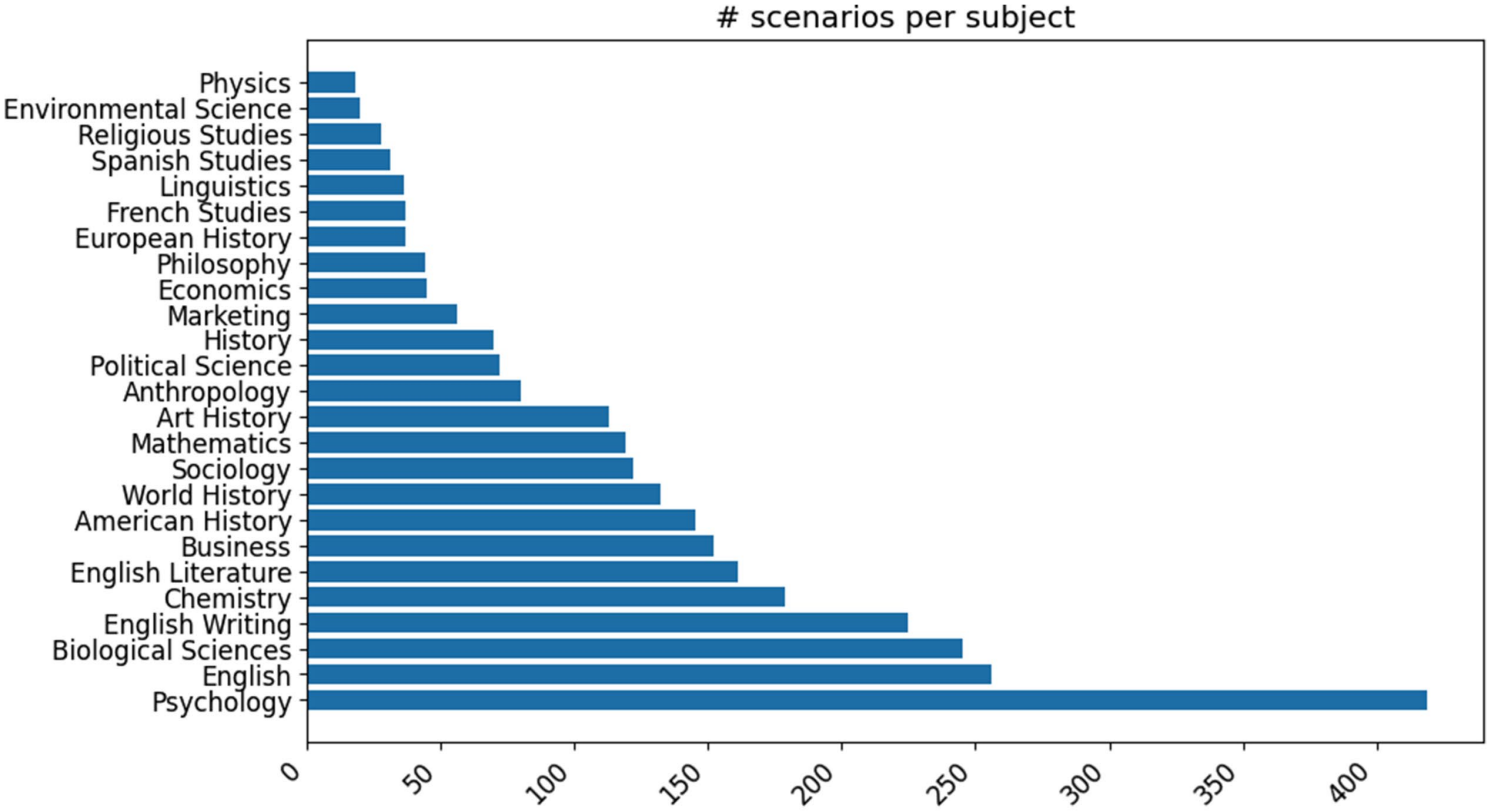
Distribution of the top 25 most common academic subjects in our original set of automatically expanded base scenarios.

One challenge we encountered with this approach is that the detailed scenarios produced by GPT-3 tended to cluster around a small number of academic subjects. We used another few-shot GPT prompt (see Appendix B) to identify any mentions of academic courses or degree programs and to identify the academic subject area(s) they belonged to. After manually reviewing and combining some closely related areas, we evaluated the distribution of the academic subjects, with the 25 most common shown in Figure 3.

We found that the top 25 most common areas accounted for over 90% of the total scenarios that featured specific academic subjects. Text generation with large language models is ultimately accomplished by repeatedly sampling from a distribution over possible words learned from their training data and further conditioned by the instructions and examples. Although we tried to modify this underlying distribution through randomly selecting and shuffling the examples we provided to condition the model’s outputs, we still found a strong preference for certain academic subjects and course names in the resulting detailed scenarios.

**Figure 4:**
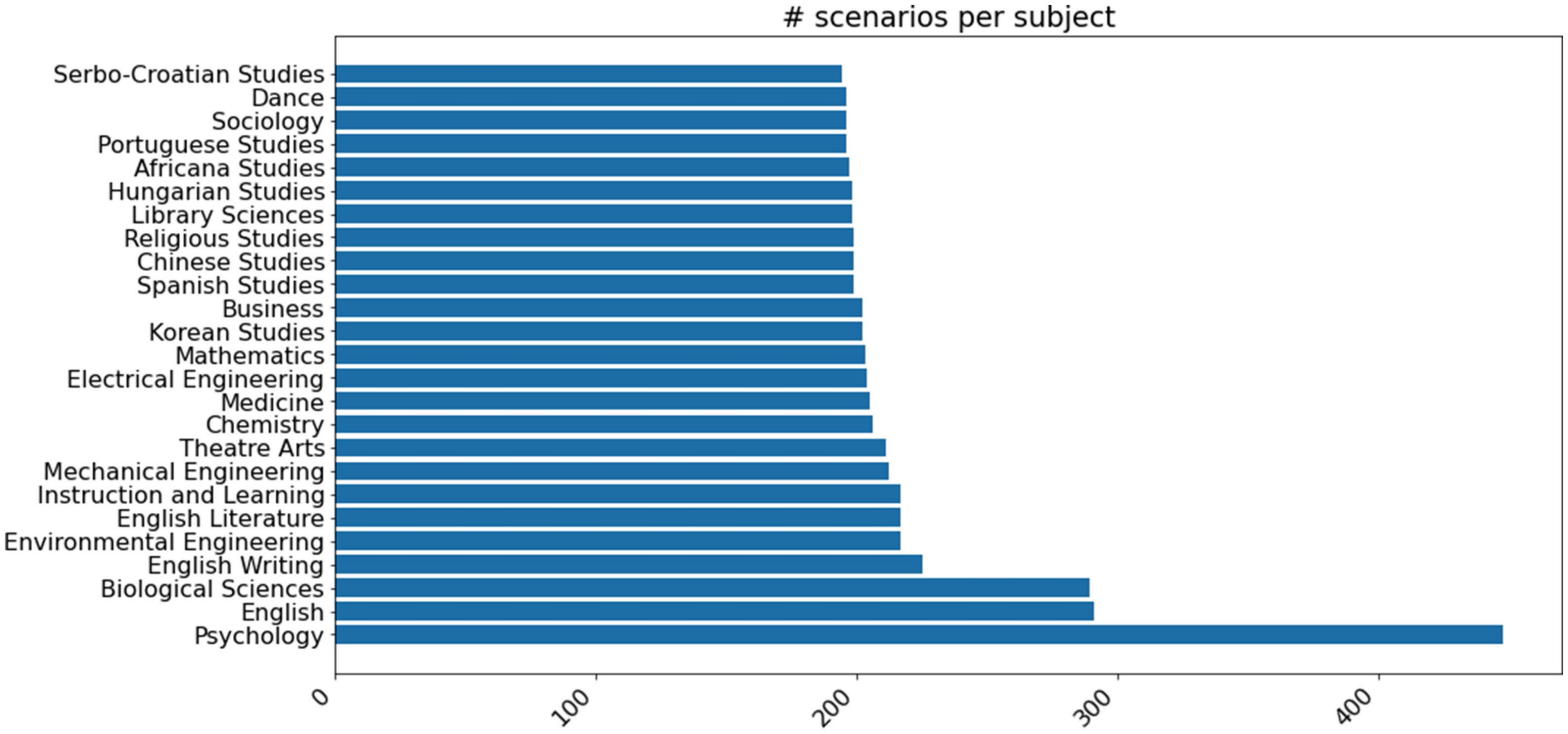
Distribution of the top 25 most common academic subjects in our set of scenarios after generating rewrites to scenarios targeting specific academic subjects.

To correct this tendency, we prompted GPT to rewrite the scenarios to focus on under-represented subject areas from a list of common university degree programs (prompt in Appendix C). For each detailed scenario with an identified academic subject, we used this prompt to rewrite the scenario to target five new subject areas. After deduplication, we ended up with an additional 14,000 scenarios, with the top 25 most common subject areas accounting for just 31% of them (see Figure 4).

### Conversation generation

To generate conversations, we created two GPT-3 prompts, one to use for generating student–student conversations and one for student-professor conversations. Each prompt consisted of three examples of scenarios and conversations as well as a simple set of instructions telling GPT-3 to include as many details from the scenario as possible in the resulting conversation (see Appendix D). We then provided a detailed scenario from our set of generated scenarios to generate new conversations. For each generated conversation, we assigned one of the student roles to be the test taker (or the only student role in the student-professor setting).

In addition, we created a third prompt with the goal of creating conversations that would require more listening from the test taker and understanding of more complex academic materials. This prompt required a broad topic such as “statistical correlation” as input, rather than a scenario, and included instructions to create conversations framed around a professor explaining the topic to a student, with the student asking questions to elicit more information. The examples used in the prompt had very long lines for the professor character. This allowed us to generate conversations that more reliably required substantially more listening from test takers compared to the first two prompts.

Using the almost 18,000 scenarios generated in the previous step and these three conversation generation prompts, we created a pool of nearly 125,000 conversations from which we sampled conversations for all subsequent experiments. When selecting conversations to use for the Interactive Listening tasks, we filtered conversations to limit the variability in the task’s requirements, including:

- 5–6 turns for the test-taker role.
- >10 words on average across all test-taker role lines.
- <40 words maximum for any non-test-taker lines.
- Test taker must have the last line in the conversation.

For the selected conversations, we additionally identified named entities in the conversations corresponding to other students or professors mentioned in the conversations. We manually reviewed the identified entities and filtered out any that were mentions of living or historical people and then replaced the names of the remaining entities with names randomly selected from a collection of the most common first and last names from countries around the world to help mitigate potential gender or regional biases in the generated conversations.

### Distractor generation

Each multiple-choice item in an Interactive Listening task corresponds to one of the test taker’s turns in the conversation. As such, the keys for the task are simply the original lines in the conversation. Extending the work of Attali et al. (2022), we obtain incorrect answers (distractors) to multiple-choice items (turns) by evaluating and selecting turns from other generated conversations.

For this work, we started by extracting each line of each conversation from the 125,000 conversations generated in the previous step, creating a bank of nearly 1 million lines of dialog. For each line, we tracked metadata related to the line’s source conversation, such as the academic topic, scenario, detailed scenario, its relative position within the conversation, and the role of the line’s speaker in the conversation.

We selected distractors for each multiple-choice item from this dialog bank using a three-step process. First, we looked at the relative position of where in the conversation the current multiple-choice item occurred. We grouped these positions into one of three categories: the first 20% of the conversation, the last 20%, or the middle 60%. We then limited candidate distractors to only those that came from the same grouped position in their source conversations. Lines at the beginning and ends of conversations feature opening and closing lines with more formulaic expressions and expectations (House and Kádár, 2023; Jucker, 2017), so this initial restriction of the distractor pool accordingly helped to eliminate distractors that would potentially be very easy to identify for test takers.

Second, we ordered potential distractor candidates based on their source conversation metadata. We gave preference to lines that came from conversations generated from the same detailed scenario, and then to those from the same base scenario. This approach is similar to the one employed in Attali et al. (2022) which used passages with similar subjects and characteristics as sources for distractors. We additionally preferred lines spoken by student characters in the conversations. At this step, we also ensured distractor candidates matched surface characteristics of the key, such as the length and ending punctuation. We also applied an automated editing step to ensure consistency of character names at this step. If a candidate distractor contained a mention of a character external to the conversation, such as a professor, then we automatically replaced the name in the distractor to match one of the names of the characters mentioned in the surrounding task conversation. We selected the top 200 candidates ordered by these criteria for further evaluations.

Finally, we computed a set of additional metrics to re-rank and select distractors for each multiple-choice item for a given task. We used the sentence-transformers library to compute vector space cosine similarity between each candidate distractor and the key. We also used a large language model to estimate how plausible the distractor would be at the given point in the conversation compared with the key.

For a given Interactive Listening task with *N* multiple-choice items, we defined *O* as the original prompt that generated the conversation, *S* as the scenario for the task, *t_i_* as the i-th turn in the conversation, *C_<i_* as the concatenation of all turns in the conversation prior to turn *i*, *k_i,n_* as the key to the n-th multiple-choice item, corresponding to *t_i_* in the conversation, and *d_i,j,n_* as the *j*-th candidate distractor for the *n*-th multiple-choice item, administered at *t_i_*.

To get the likelihood of the key for the *n*-th multiple-choice item, we concatenated *O, S, C_<i_*, *k_i,n_*, and *t_i + 1_*. We submitted this input, *I*, to OpenAI’s text-babbage-002 model and had it return the log probabilities for each token *m_h_* in the input, $\forall_{m\in I}\log\left(P\left(m\right)\right)$ . We then computed the token-average log probability metrics for the key, *k_i,n_* and the turn that followed it, *t_i + 1_*:


$$\begin{array}{l} key_{i,n}\_\log\_\mathrm{probability}=\frac{1}{len_{tk}\left(k_{i,n}\right)}*\\ \;\underset{m_{h}\in k_{i,n}}{\sum }\log\left(P\left(m_{h}|O,S,C_{<i},m_{<h}\right)\right) \end{array}$$

$$\begin{array}{l} \mathrm{post}\_key_{i,n}\_\log\_\mathrm{probability}=\frac{1}{len_{tk}\left(t_{i+1}\right)}*\\ \;\underset{m_{h}\in t_{i+1}}{\sum }\log\left(P\left(m_{h}|O,S,C_{<i},k_{i,n},m_{<h}\right)\right) \end{array}$$

We can repeat this process for each distractor candidate *d_i,j,n_*, concatenating *O*, *S*, *C_<i_*, *d_i,j,n_*, and *t_i + 1_* and compute:


$$\begin{array}{l} \mathrm{distracto}r_{i,j,n}\_\log\_\mathrm{probability}=\frac{1}{len_{tk}\left(d_{i,j,n}\right)}*\\ \;\underset{m_{h}\in d_{i,j,n}}{\sum }\log\left(P\left(m_{h}|O,S,C_{<i},m_{<h}\right)\right) \end{array}$$

$$\begin{array}{l} \mathrm{post}\_\mathrm{distracto}r_{i,j,n}\_\log\_\mathrm{probability}=\frac{1}{len_{tk}\left(t_{i+1}\right)}*\\ \;\underset{m_{h}\in t_{i+1}}{\sum }\log\left(P\left(m_{h}|O,S,C_{<i},d_{i,j,n},m_{<h}\right)\right) \end{array}$$

When selecting distractors for the n-th multiple-choice item, we evaluated the difference between each $\mathrm{distracto}r_{i,j,n}\_\log\_\mathrm{probability}$ and $key_{i,n}\_log\_\mathrm{probabillity}$ as an estimate of how likely the distractor is in the context of the conversation, relative to the key. A positive difference meant that the model estimated that the distractor is more likely than the key, which indicated that the distractor could be a valid answer in a multiple-choice item. We likewise measured the difference between the $\mathrm{post}\_\mathrm{distracto}r_{i,j,n}\_\log\_\mathrm{probability}$ and $\mathrm{post}\_key_{i,n}\_\log\_\mathrm{probability}$ , which estimated whether the distractor ultimately could still fit in with the remainder of the conversation. A positive or near-0 difference here could serve as additional evidence that a distractor fits in potentially too well with the context of the conversation. For multiple choice items that used the final turn in the conversation as the key, the *post_key/distractor* metrics were not computed as they lacked a following turn.

We set thresholds on the two log probability difference metrics and the cosine similarity metric to filter out distractors that were too plausible in the context of the conversation or too similar to the key. We then sorted distractors by the ratio between their key-distractor log probability difference and their cosine similarity to the key. This resulted in a ranking that prioritized distractors that maintained a balance between how similar they were to the key and how likely they were in the context of the conversation, with distractors that were too unlikely or too dissimilar ending up near the bottom of the rankings. We finally selected the top K candidates for review and piloting, with the added requirement that each candidate had a cosine similarity less than or equal to 0.6 with all other selected candidates to avoid having multiple, highly similar distractors for a single item.

### Task design pilots

This section presents the results from a set of experiments conducted to support iteration on the design of the Interactive Listening task and gather evidence about its performance. We conducted these experiments using a piloting platform that was developed as part of the DET practice test. This practice test is an online version of the DET, freely available to anyone interested in the test, with over 10,000 test sessions daily. Similar to the operational test, the practice test is fully adaptive and test takers have the opportunity to respond to and practice all of the tasks that are included in the operational test. The piloting platform is an opt-in section at the end of the practice test with experimental tasks, and around 50% of practice test takers choose to complete these additional experimental tasks. We used this platform to conduct around 10 controlled experiments over the course of 6 months to test different task design options.

We briefly report the results of three of these experiments, exploring the effects of (1) the number of multiple-choice options, (2) displaying the full history of the conversation, and (3) allowing replay of the interlocutor’s audio on test taker performance.

In these experiments, we focused on item statistics and response times. Item difficulty was measured using percent correct and item discrimination was measured using item-total correlations where total scores were practice test scores.

### Number of options

We start with results from an experiment that varied the number of options, comparing 4 options (3 distractors) with 5 options. We used a set of 29 Interactive Listening tasks with a total 150 multiple-choice items and collected responses from at least 250 test takers for each conversation in each condition. As expected from the decreased opportunity for guessing, items with five options ( M=0.54 , SD=0.14 ) were harder than items with four options ( M=0.57 , SD=0.14 ). A paired t-test confirmed that this difference is significant ( $t\left[149\right]=2.55$ , p=.012 ), although the effect size is quite small ( d=0.19 ) (Cohen, 1988).

**Figure 5:**
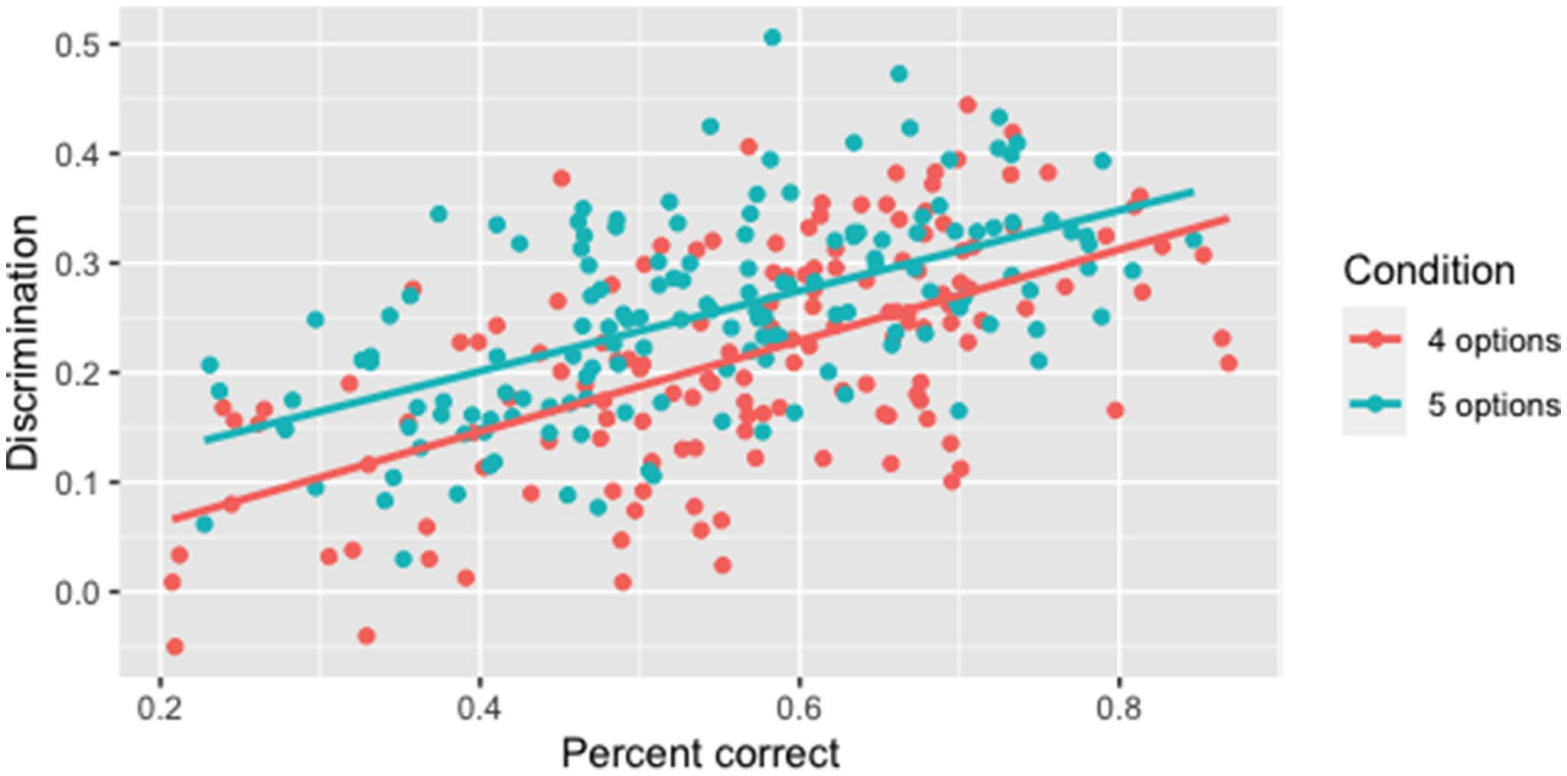
Results from varying number of options: Discrimination as a function of difficulty.

Similarly, five options ( M=0.25 , SD=0.09 ) yielded higher discrimination than four options ( M=0.22 , SD=0.10 ). A paired t-test confirmed that this difference is significant, ( $t\left[149\right]=-4.32$ , p<0.001 ), with a medium effect size ( d=0.38 ). Figure 5 shows that the advantage in discrimination held after controlling for item difficulty, indicating that having five options in an item is more discriminating than four options regardless of item difficulty.

**Figure 6:**
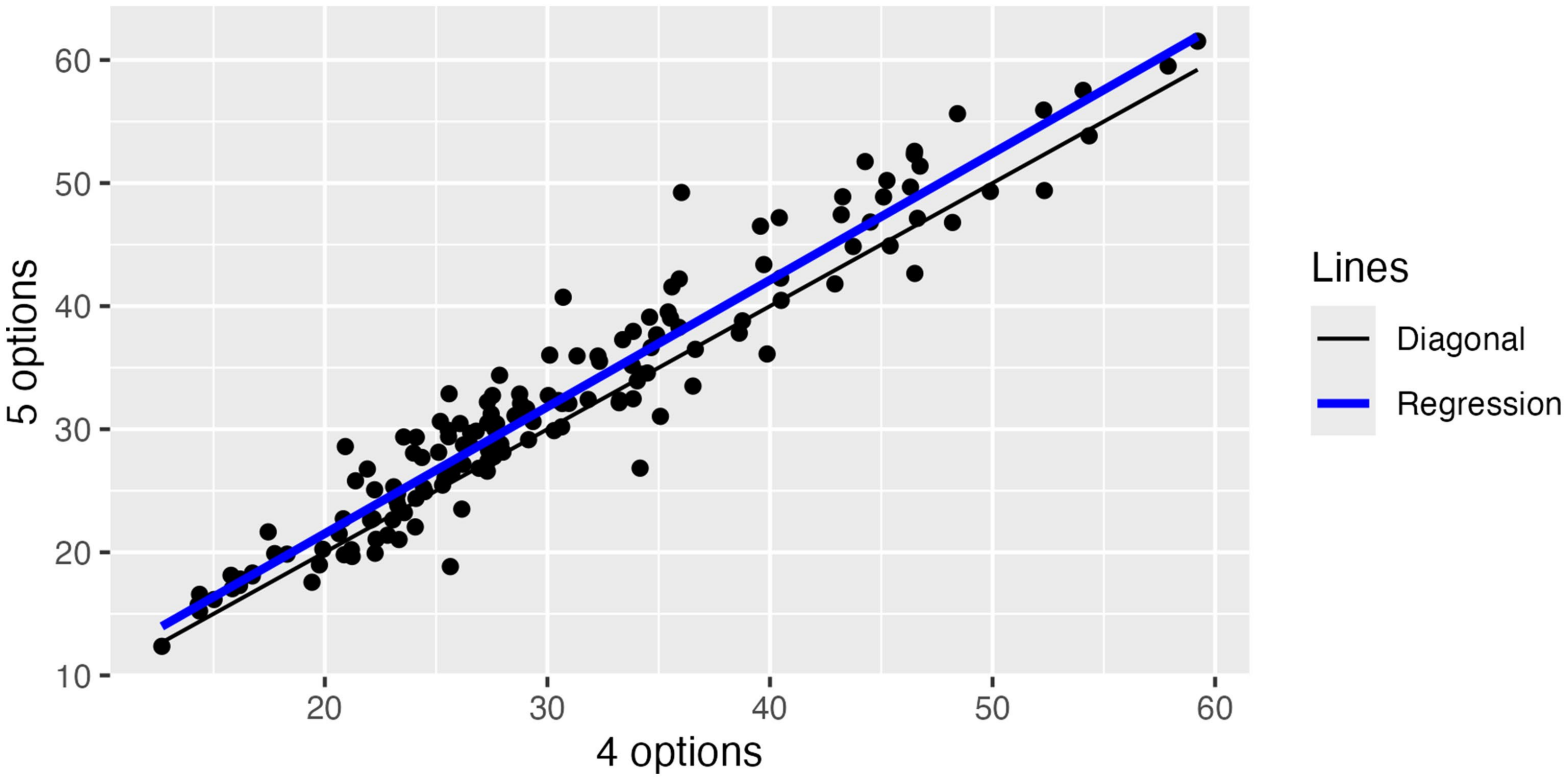
Results from varying the number of options: Median response times.

Finally, Figure 6 shows that median response times with 5 options are only 2 s longer than with 4 options.

As a result of this experiment, we decided to continue with 5 options for higher psychometric standards.

### Showing history

The second experiment tested whether the availability of the history of the conversation had any impact on the test-taker performance. In one condition, test takers were able to review the full history of the conversation, including text transcripts for each of the interlocutor’s prior turns. In the other, test takers could only review a transcript of the interlocutor’s most recent turn, as well as their own most recent response. The motivation for allowing test takers to review the full history of the conversation was to minimize the potential for construct-irrelevant variance from the effect of memory on test-taker performance. We used the same set of 29 tasks from the previous experiment and collected responses from 250 test takers for each. We found that showing the history had very small and non-significant effects on median response time (means of 36 s in both conditions), item difficulty (means of 54% correct in both conditions), and item discrimination ( M=.239 with no history and M=.246 with history). We ultimately decided to show history to test takers as doing so would give us more flexibility in the future to increase the length of the conversations, both in the number of turns and the maximum length of each turn, without the possibility of memory interfering with test-taker performance.

### Number of plays

The last experiment manipulated the number of allowed plays for each interlocutor turn in the conversation: a single play (no replay allowed) vs. two plays (one replay allowed). The motivation for not allowing replays was to more closely simulate a real conversation. This experiment was based on a subset of 10 of the conversations from prior experiments with a total of 51 turns and 250 responses each. In the replay condition, we found that test takers only used the feature in 16% of the total items. The effect of allowing a replay on item statistics was analyzed using paired t-tests. Allowing a replay had a small effect on median response time in seconds ( $\Delta M_{rt}=1.29$ , p<.001, d=0.14 ), a small effect on percent correct ( $\Delta M_{rt}=0.02$ , p=0.001 , d=0.15 ), but no effect on discrimination ( $\Delta M_{rt}=0.00$ , p=.776 , d=−0.03 ). Because the replay feature was not extensively used and had minor effects on item statistics, we decided to allow only a single play, with an added advantage of simplifying the user interface.

### Large-scale pilot

The culmination of the iterative task development process was a large-scale pilot in which the task was administered as a regular part of the DET practice test. The purpose of the pilot was to evaluate the quality of the AIG processes described in the previous section from both a human review and psychometric perspective. We also wanted to assess the psychometric properties of the overall task within the context of a larger assessment, as opposed to the opt-in process used in the previous section.

In particular, the questions we wanted to answer with this pilot were:

1. What is the quality of the automatically generated conversations and multiple-choice items, as assessed by expert human reviewers?
2. To what extent do linguistic and semantic features of the items impact their psychometric properties?
3. To what extent do the amounts of audio and text content impact psychometric properties?
4. To what extent do human review processes improve the psychometric properties of the AIG items?
5. To what extent does the multi-turn format of the task impact the psychometric assumption of local item dependence?

### Pilot item bank creation

To create the item bank for the pilot, we sampled 900 conversations from the set of 125,000 we generated, distributed across all of our base scenarios and academic subjects. Each of the test taker turns was converted into a multiple-choice item for which 6 distractors were generated. In practice, some items had fewer than 6 distractors, typically due to a combination of the key being extremely short or extremely long as well as our other filters and restrictions. Each item then underwent an extensive human review and editing process, described in the next section.

### Human review

Our human review process was designed to ensure that we maintain high quality standards (Saville and McElwee, 2021). All tasks underwent item quality, fairness and bias (FAB), and audio quality reviews. These reviews were conducted by 25 external reviewers and six internal Duolingo team members. External reviewers had diverse backgrounds with regard to gender identity, age, and racial/ethnic background. All had at least a bachelor’s degree (and in some cases a Ph.D.) in linguistics, language studies, or a related field. All had expertise in teaching and assessing in relevant language and cultural contexts.

Each task first went through a two-phase item quality review process. The first phase was conducted by 10 reviewers, who reviewed the task, including scenario, conversation, and items. The reviewers verified that the scenarios included sufficient context, introduced the participants, and framed the dialog appropriately to ensure that the test taker would be able to successfully participate in the conversation. For the conversations, reviewers evaluated the cohesion, clarity, and logical consistency throughout the text, also ensuring that each conversation included a speech situation, a speech event, and a speech act. The general purposes of the conversations were making a request, clarifying information, gathering or sharing information, and making a recommendation. Reviewers also confirmed that the conversations met the intended purpose of the task. For the items, reviewers judged the viability of each option by ensuring that the correct answer was correct and the distractors were incorrect. As part of this process, reviewers selected the best 4 distractors out of the 6 available options and made additional edits as needed to ensure clarity, grammaticality, and topic similarity to the key. Reviewers prioritized answer options that would require the test takers to demonstrate an understanding of social dynamics such as politeness and indirectness, particularly in relation to social distance factors. In cases where there were fewer than four available distractors, reviewers were instructed to write new distractors. Item reviewers were also trained on fairness guidelines and edited out any content that clearly violated these guidelines. All reviewer edits then went through a second phase, wherein the edits from the first phase were carefully reviewed, evaluated, and incorporated by a team of four expert reviewers. If a task required extensive edits, defined as requiring more than 20 min to fix, then reviewers abandoned the task. 725 out of 900 tasks (81%) successfully passed the item quality review.

In analyzing the edits that reviewers made, the majority aimed to address issues such as:

- Content that clearly violated our fairness guidelines.
- Items that required background knowledge about a specific topic to answer.
- Overly complex topics and technical jargon.
- Logical inconsistencies.

Throughout the review we refined our item review guidelines in response to inquiries from item reviewers. For example, discussions about whether certain academic terms, such as internship, were globally relevant, or considering whether conversations about receiving a bad grade could potentially cause test-taker anxiety.

Following the item quality review, expert FAB reviewers carefully evaluated the scenarios, conversations, and options for any remaining content that could be controversial, too culturally specific, or unfamiliar to the intended global test taker audience. Each piece of content was given a pass or fail decision by expert FAB reviewers, and all tasks were triple reviewed. 719 out of 725 (99%) of the conversations passed the FAB review.

We additionally reviewed the audio quality of the TTS for the interlocutor’s turns. 477 out of 3,245 turns (15%) needed revision due to inappropriate pronunciation, word or sentence-level stress, and distracting or incorrect intonation. Edits to the TTS turns were completed by internal Duolingo team members.

In summary, following all reviews and adjudication a final set of 713 out of 900 tasks (79%) were retained. Overall, each task was reviewed by 6–7 people and the review process took about 1 h per task across all reviews. The median time per task in each review phase was 25 min (single review) for item review phase one, 9 min (single review) for item review phase two, 9 min (across three reviews) for FAB review, 4.5 min (single review) for audio review, and 8 min (single review) for final edits needed for around half of the tasks.

### Pilot task administration

The large-scale pilot of the Interactive Listening task was administered as part of the DET practice test. At the end of the practice test, test takers were randomly assigned two of the 713 conversations, one student–student and one student-professor conversation. The time limit for each conversation was 4 min. The pilot was active for 15 days, during which 167 thousand sessions were completed (almost all of them with two conversations), with an average of 464 sessions per conversation.

## Results

### Predictors of item difficulty and discrimination

We first look at how different features of the multiple-choice items impact their difficulty (percent correct) and discrimination (item-total correlation). For each question, we extracted a combination of features similar to those used in the distractor generation process, as well as edit-based features from the results of the human review process. For measures related to distractors, we averaged the values across all distractors after comparing several aggregation strategies and finding no differences. Specifically, these features were:

- Key log probability.
- Distractor log probability.
- Difference between distractor and key log probabilities.
- Cosine similarity between the key and each distractor.
- Length-normalized edit distance for the key.
- Length-normalized edit distance for each distractor.
- Ratio of the character length of each distractor to the key.

We conducted a regression analysis on percent correct using these features as predictors. We found that the log probability difference feature had high correlation with distractor log probability, as most keys fall within a narrow range of log probabilities, so we removed the difference feature from our analysis. Table 1 summarizes the regression results of the remaining standardized predictors on percent correct ( $R^{2}=.11$ ).

**Table 1:** Regression results for percent correct with standardized predictors.

Predictor	*b*	*t*	*p*
Intercept	0.68	280.93	<0.001
Key LP	0.06	19.04	<0.001
Distractor normalized edit distance	0.00	−0.15	0.877
Key normalized edit distance	0.02	7.89	<0.001
Distractor LP	−0.04	−12.65	<0.001
Distractor key similarity	0.00	−1.16	0.248
Distractor key length ratio	−0.03	−10.16	<0.001

In this analysis, the log probability (LP) of the key had the largest effect on difficulty, with greater LP leading to easier items, while the LP of distractors had the second largest (negative) effect on easiness. These results confirm that the log probabilities from large language models at least partially capture the plausibility of a line in a conversation and as a result could be used to make the items easier or more difficult by selecting keys and distractors based on their log probabilities.

We also found that larger edits to keys led to easier items, but distractor edits do not have a significant effect. This suggests that human reviewers may have a tendency to remove elements that make items less clear, tricky, or otherwise hard.

Finally, distractors that were longer than the key led to more difficult items, suggesting that test takers tend to gravitate toward longer options.

For question discrimination, a corresponding regression analysis included percent correct as an additional predictor, since percent correct alone has a strong effect on discrimination ( $R^{2}=.28$ ), with easier items associated with higher discrimination. The full regression model that included all other predictors had only slightly higher predictive power than the preceding model ( $R^{2}=.29$ ). The LP of keys and distractors, along with distractor edits, were shown to be significant predictors of item discrimination ( p<.05 ), but none of them had standardized coefficients that were larger than 0.005.

### Audio and text length

Since the Interactive Listening task requires both listening to the interlocutor’s turns and reading the options to respond, we analyzed the effect of listening load (length of previous turn’s audio) and reading load (total number of characters across options) on item difficulty and discrimination to ensure that there was as little interference from reading as possible in the assessment of interactional competence.

Regression results of the standardized load predictors on percent correct ( $R^{2}=.03$ ) indicated that only listening load ( b=−0.03 , 95% CI $\left[-0.03,-0.02\right]$ ) had a significant effect, with a higher listening load leading to less easy items, as intended.

Regression results of the standardized load predictors and percent correct on discrimination ( $R^{2}=.27$ ) indicated that both listening load ( b=0.00 , 95% CI $\left[0.00,0.00\right]$ ) and reading load ( b=0.00 , 95% CI $\left[0.00,0.00\right]$ ) were not significant.

### Impacts of edits on distractors

We next look at how the changes made by reviewers impact distractor attractiveness and discrimination. Attractiveness was computed as the proportion of test takers selecting that particular distractor; discrimination was computed as a point-biserial whereby only test takers who selected either the distractor or the correct answer were considered (Attali and Fraenkel, 2000). We measured the normalized character edit distance between the original and revised versions of the distractors and discretized the values into 4 categories based on manual assessment of the significance of the edits those values represent. We separately grouped the small number of human-generated distractors into their own category. The results are shown in Table 2. We found that the small number of completely human-generated distractors were slightly more discriminating and attractive than the rest of the distractors, and that for the rest, more heavily edited distractors tended to be more discriminating and less attractive.

**Table 2:** Distractor attractiveness and discrimination by normalized character edit distance range.

		Discrimination		Attractiveness	
Edit distance	*N*	*M*	SD	*M*	SD
Human-generated distractors	202	−0.213	0.088	0.086	0.087
No change, 0 edit distance	6,870	−0.194	0.088	0.082	0.076
Slight change, 0.01–0.25	3,033	−0.195	0.084	0.084	0.074
Medium change, 0.25–0.50	2,293	−0.199	0.084	0.076	0.072
Large change, 0.50–0.99	2,284	−0.206	0.083	0.066	0.065

However, in a regression analysis on attractiveness controlling for all other distractor metrics that appeared in Table 1 ( $R^{2}=.08$ ), the effect of the edit distance was not significant ( $t\left(14674\right)=-1.29$ , p=.198 ). Similarly, in a regression analysis on discrimination controlling for all other metrics ( $R^{2}=.01$ ), the effect of edit distance was not significant ( $t\left(14674\right)=-1.64$ , p=.101 ). These regression analyses also show that all other metrics are also very weak predictors of distractor performance. This was expected, as these metrics were used to select distractors and would therefore likely show a weaker relation with distractor performance measures due to restriction of range.

### Reviewer effects on item easiness

**Figure 7:**
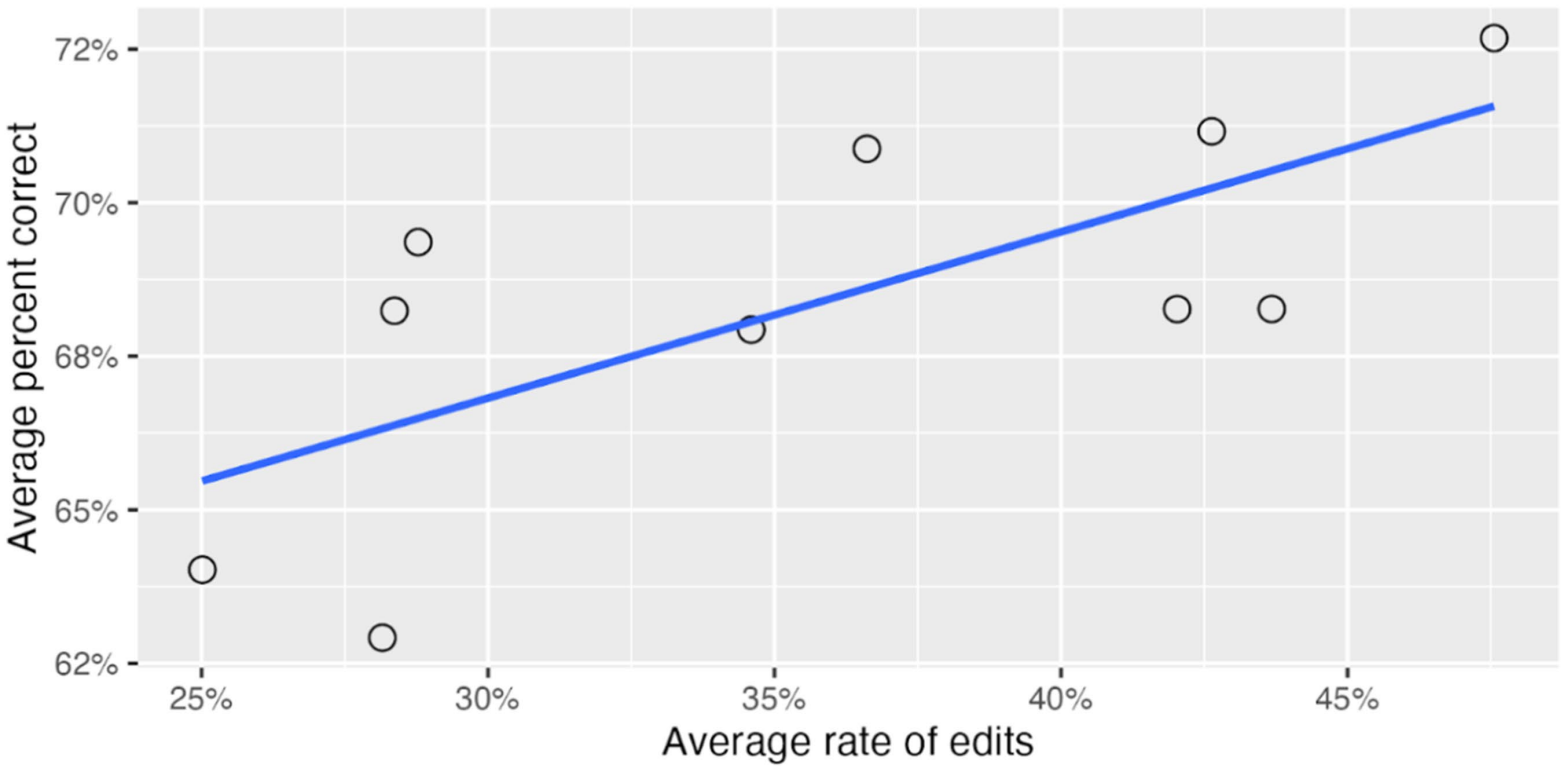
Effect of per-reviewer rate of edits on average item easiness.

Across the 10 reviewers of the initial machine-generated conversations and items, we found large inter-reviewer differences in the amount of edits to the content. These differences accounted for 59% of the variance in total edits to the conversation turns (keys and distractors). In other words, reviewers differed greatly in how much they edited an item (see Figure 7). As a result, we wanted to assess whether these differences in the tendency to revise machine-generated content have downstream effects on the psychometric properties of items–whether the more an item is edited, the easier it becomes. This in turn could help us improve our guidance to reviewers about how much to revise the items or help identify particularly effective editing patterns among our reviewers.

In a mixed-effects analysis predicting percent correct from the average rate of edits for reviewers, the reviewer random effect explained just 2.3% of the variance in question easiness. However, the average rate of edits per reviewer was a significant predictor of question easiness ( p=.03 ). Figure 7 presents, for each of the 10 reviewers, their average rate of edits across all questions and the average percent correct of these questions. It shows a high correlation between rate of edits for each reviewer and the easiness of the edited items ( r=.71 ).

**Figure 8:**
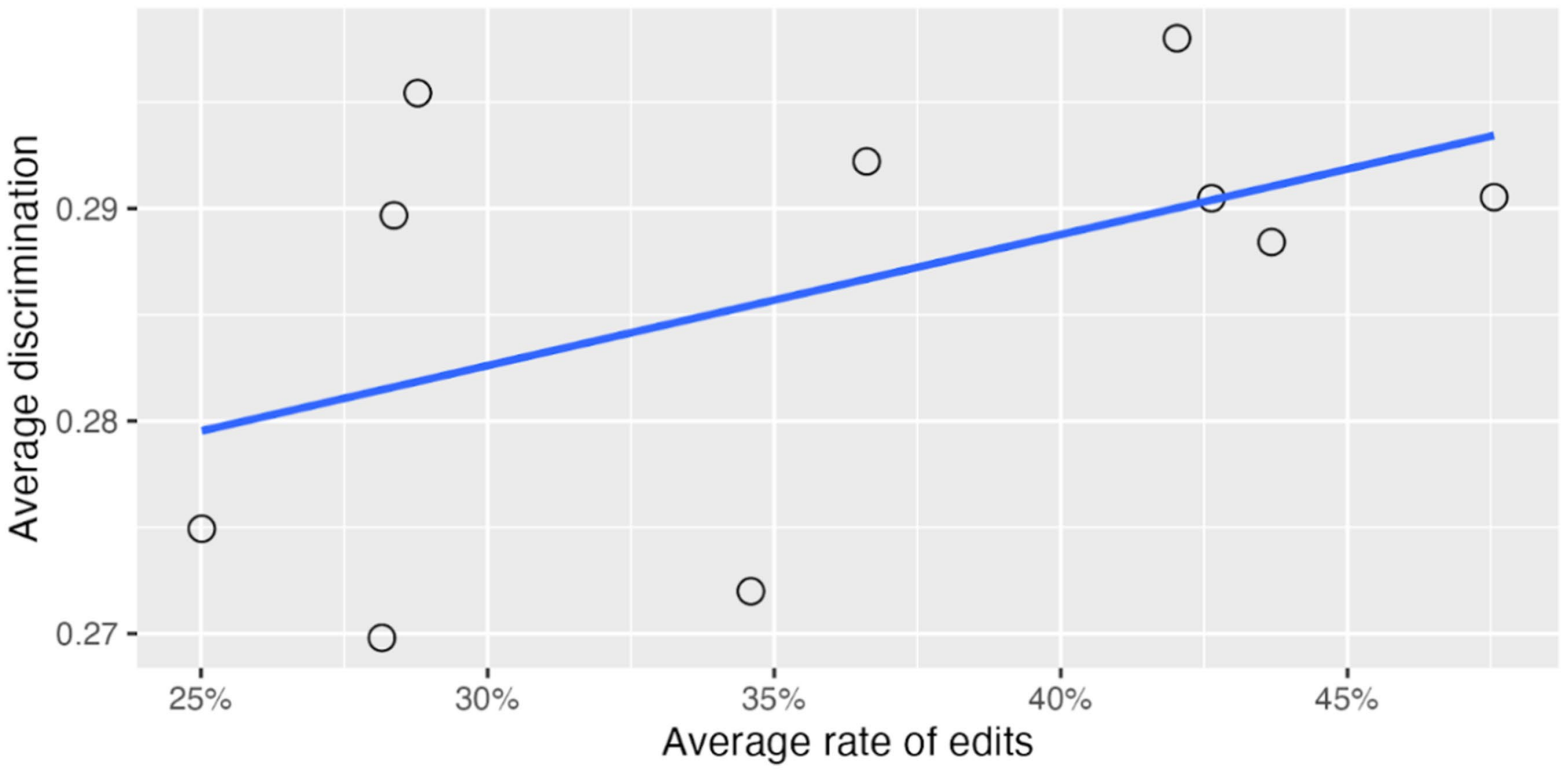
Effect of reviewer rate of edits on average question discrimination for a 68% correct item.

Similarly, in a mixed-effects analysis predicting discrimination from percent correct and the average rate of edits for reviewers, the reviewer random effect explained just 1.8% of the variance in question discrimination. In this model the average rate of edits per reviewer was not a significant predictor of discrimination ( p=.21 ). Figure 8 shows the relation between the average rate of edits for each reviewer and average discrimination for a typical percent correct of 68%.

In summary, reviewers differed greatly in how much they edited the machine-generated content they reviewed, ranging from 25 to 48%. These differences accounted for about 2% of the variance in both easiness and discrimination of items, with higher rate of edits associated with easier items.

### Local item dependence

An important assumption for psychometric analysis of test items is that the dependency between responses to any pair of items is due only to the trait being measured. Pairs of items that violate this assumption are said to exhibit local item dependency (LID). The presence of LID is problematic in that it lowers the psychometric usefulness of items. It is well known that sets of items that are based on a common stimulus, such as reading and listening comprehension items, can result in local dependence because the information used to answer different items is interrelated in the stimulus (Yen, 1993). In the present context, the threat of LID is even greater since the items represent successive turns in a single conversation and moreover, test takers receive feedback about their previous answers.

A standard IRT approach for investigating LID between test items is to compute the correlation between residual responses – the difference between the expected model-based score and the actual observed score (Yen, 1984). As an approximation of this approach, we computed the partial correlations between pairs of items, controlling for total practice test score. Over all 7,681 pairs of items, partial correlations were generally quite low ( M=0.13 , SD=0.08 ), and only 3.6% of pairs exceeded a common threshold of 0.3 (Christensen et al., 2017) for categorizing residual correlations as indicating LID. Moreover, the difference between residual correlations of adjacent pairs ( M=0.15 , SD=0.09 ) and non-adjacent pairs ( M=0.13 , SD=0.08 ) was very small.

## Discussion

In this paper we describe the creation of a novel approach to expand measurement of the construct of listening to include more elements of interactional competence, such as a more evolved purpose of listening, the awareness of variation in the language against different purposes of communication and different interlocutors, and conversation management across several turns. The design of the task sets it apart from traditional measures as the purpose of listening moves beyond comprehension and into purposes for communication that are more directly related to the TLU domain (e.g., asking clarification questions in office hours and planning a study group; Aryadoust and Luo, 2023). Providing immediate feedback about test takers’ choices (Attali and Powers, 2010) is another unique design decision in this task that enables its interactive nature and allows test takers to engage as active participants in a multi-turn conversation. The item bank consisting of different situations with interlocutors of varying power distance also addresses limitations in measuring interactional competence that are introduced by human interlocutors in speaking tasks (Galaczi and Taylor, 2018). Organizing the task around scenarios with different types of interlocutors allows for the introduction of language variation that aligns with variability in authentic interactions resulting from changes in power dynamics between interlocutors, purposes for communication, and topics. This task also extends measurement of the construct beyond traditional discourse completion tasks, which usually require test takers to select or provide a single turn of a conversation and are often limited to the beginning or end. In the Interactive Listening tasks, test takers need to show the ability to open a conversation, close a conversation, understand the interlocutor, and advance the conversation toward a specific purpose across several consecutive turns. As a result, it measures the ability to interact through an entire conversation while retaining the psychometric usefulness of the items despite the potential threat of local item dependence.

We provide supporting evidence for using NLP indices to generate, filter, and select keys and distractors. LLM-based log probability was shown to be a useful metric for manipulating item easiness, with keys with higher log probabilities yielding higher item easiness and distractors with higher log probabilities yielding lower item easiness. These metrics were not as useful when predicting discrimination, suggesting that item discrimination is a harder concept to model for AIG. This nonetheless illustrates the potential for AIG to target a specific range of item difficulty at the time of item generation, allowing for a construction of large item banks spanning a wide range of difficulty that is needed for computer adaptive tests.

Human review of the generated items showed that our approaches to generating content and items for this task are generally successful with room for improvement. Revisions by experts led to measurable improvements in the quality of the resulting items. Completely human-written distractors were most discriminating and attractive, with human reviewers providing edits to the keys that potentially clarify them further. This provides support for the human-in-the-loop approach to item generation and review, where human expertise informs AIG (and vice versa) to produce quality items. Patterns in reviewer edits can be identified and incorporated in later rounds of AIG to produce items that obviate the need for heavy human edits, reducing the amount of human effort required to produce high-quality items.

The task design is not without limitations. While it is a direct measure of listening in conversational settings, it is an indirect measure of speaking ability and of interactional competence as test takers need to understand the conversation and its goals to progress successfully through the task. Test takers select their responses from a list of options instead of speaking their responses directly. The results from the large-scale pilot, however, show that test-taker performance was not modulated by the amount of reading involved. When taken as a whole, the task extends the listening construct from listening for the purpose of comprehension to listening for the purpose of communication.

## Future directions

### Attribute-driven conversation generation

This work used GPT-3 to generate the conversations used for the Interactive Listening tasks which was, at the time of development, the most powerful commercially available LLM. Since then, many new open-source (Jiang et al., 2023; Touvron et al., 2023) and commercially available (Anthropic, 2024; OpenAI et al., 2024) LLMs have been released with significantly improved performance across a wide range of tasks. Most relevant to our work is the improved ability to generate content according to an extensive set of descriptors (Lynch et al., 2023) as well as the ability to model personality traits in its outputs (Jiang et al., 2024). These capabilities can enable generation of conversations that target more specific attributes of the interlocutors, such as the relationship between the two, or include a wider range of elements and details that make the generated conversations more varied and distinctive. We have begun experimenting with these more complex prompt structures and found that they significantly improve the coherence, diversity, and authenticity of the generated conversations.

### Dynamic interactions

Advances in LLMs also open up the possibility of having live interlocutors in assessments of interactional competence. Prior work on dialog systems to identify issues such as dialog breakdown (Higashinaka et al., 2016), models fine-tuned to play specific roles or model personality traits (Han et al., 2024), and work designed to keep fine-tuned LLMs aligned with safety goals (Lyu et al., 2024) can all be leveraged to build reliable, safe systems. Additionally, there are active research communities exploring ways to mitigate the risks posed by hallucinations in LLM-generated output (Ji et al., 2023), as well as jailbreaking (Kumar et al., 2024; Xie et al., 2023), which are necessary for any application which allows direct user interaction with an LLM. As these different areas of research continue to develop, the use of dynamic, test-taker driven interactions as part of a high-stakes assessment becomes more feasible.

### New task and item formats

We focused on short conversations between two participants centered around academic contexts. The design of the task is flexible and could easily be modified to involve more than 2 participants in order to assess interactional competence in group settings or feature longer conversations depending on the needs of the assessment. The scenario-based content generation process can also be easily extended to non-academic contexts, such as service encounters or occupational settings.

As generative AI continues to develop at a rapid pace, assessment developers can look to use these developments in technology to create language assessment tasks that address current limitations while maintaining and improving the validity of tasks.

## Data Availability

The original contributions presented in the study are included in the article/Supplementary material, further inquiries can be directed to the corresponding author.

## Footnotes

^1^The most recent commercially available version of GPT at the time of development (Fall, 2022).

^2^This model has been replaced by the more recent text-davinci-003 model. See https://platform.openai.com/docs/deprecations for more details.

^3^http://sbert.net, using model paraphrase-MiniLM-L6.

## References

[ref2] Anthropic. (2024). Introducing the next generation of Claude. Available at: https://www.anthropic.com/news/claude-3-family (Accessed July 15, 2024).

[ref3] Aryadoust V. Luo L. (2023). The typology of second language listening constructs: a systematic review. Lang. Test. 40, 375–409. doi: 10.1177/02655322221126604

[ref4] Aryadoust V. Zakaria A. Jia Y. (2024). Investigating the affordances of OpenAI’s large language model in developing listening assessments. Comput. Educ. 6:100204. doi: 10.1016/j.caeai.2024.100204

[ref5] Attali Y. (2018). “Automatic item generation unleashed: an evaluation of a large-scale deployment of item models” in Artificial intelligence in education. eds. Rosé C. P. Martínez-Maldonado R. Hoppe H. U. Luckin R. Mavrikis M. Porayska-Pomsta K. (Cham: Springer International Publishing), 17–29.

[ref6] Attali Y. Fraenkel T. (2000). The point-biserial as a discrimination index for distractors in multiple-choice items: deficiencies in usage and an alternative. J. Educ. Meas. 37, 77–86. doi: 10.1111/j.1745-3984.2000.tb01077.x

[ref7] Attali Y. Powers D. (2010). Immediate feedback and opportunity to revise answers to open-ended questions. Educ. Psychol. Meas. 70, 22–35. doi: 10.1177/0013164409332231 PMC5965532 29795888

[ref8] Attali Y. Runge A. LaFlair G. T. Yancey K. Goodwin S. Park Y.. (2022). The interactive reading task: transformer-based automatic item generation. Front. Artif. Intell. 5:903077. doi: 10.3389/frai.2022.903077, PMID: 35937141 PMC9354894

[ref9] Bartram D. Hambleton R. (2005). Computer-based testing and the internet: Issues and advances. Hoboken, NJ: John Wiley and Sons.

[ref10] Bejar I. I. (2002). “Generative testing: from conception to implementation” in Item generation for test development (Mahwah, NJ: Lawrence Erlbaum Associates Publishers), 199–217.

[ref11] Belzak W. C. M. Naismith B. Burstein J. (2023). “Ensuring fairness of human-and AI-generated test items” in Artificial intelligence in education. Posters and late breaking results, workshops and tutorials, industry and innovation tracks, practitioners, doctoral consortium and blue sky. eds. Wang N. Rebolledo-Mendez G. Dimitrova V. Matsuda N. Santos O. C. (Cham: Springer Nature Switzerland), 701–707.

[ref001] Bezirhan U. von Davier M. (2023). Automated reading passage generation with OpenAI’s large language model. Comput. Educ.: Artif. Intell. 5:100161. doi: 10.1016/j.caeai.2023.100161

[ref12] Bolender B. Foster C. Vispoel S. (2023). The criticality of implementing principled design when using AI technologies in test development. Lang. Assess. Q. 20, 512–519. doi: 10.1080/15434303.2023.2288266

[ref13] Brown J. D. (2018). “Assessing pragmatic competence” in The TESOL encyclopedia of English language teaching. ed. Liontas J. (Hoboken, NJ: Wiley), 1–7.

[ref14] Brown T. Mann B. Ryder N. Subbiah M. Kaplan J. D. Dhariwal P.. (2020). “Language models are few-shot learners” in Advances in neural information processing systems. eds. Larochelle H. Ranzato M. Hadsell R. Balcan M. F. Lin H. (Virtual: Curran Associates, Inc.), 1877–1901.

[ref15] Buck G. (2001). Assessing Listening. Cambridge, UK: Cambridge University Press.

[ref16] Carr N. (2011). Designing and analyzing language tests: A hands-on introduction to language testing theory and practice. Oxford, United Kingdom: Oxford University Press.

[ref17] Chen M. Papangelis A. Tao C. Kim S. Rosenbaum A. Liu Y.. (2023). “PLACES: prompting language models for social conversation synthesis” in Findings of the Association for Computational Linguistics: EACL 2023. eds. Vlachos A. Augenstein I. (Dubrovnik, Croatia: Association for Computational Linguistics), 814–838.

[ref18] Chen M. Papangelis A. Tao C. Rosenbaum A. Kim S. Liu Y.. (2022). *Weakly supervised data augmentation through prompting for dialogue understanding*. In: NeurIPS 2022 workshop on synthetic data for empowering ML research.

[ref19] Chen J. Yang D. (2021). “Simple conversational data augmentation for semi-supervised abstractive dialogue summarization” in Proceedings of the 2021 Conference on Empirical Methods in Natural Language Processing. eds. Moens M.-F. Huang X. Specia L. Yih S. W. (Online and Punta Cana, Dominican Republic: Association for Computational Linguistics), 6605–6616.

[ref20] Chiu E. Lenzo K. Swecker G. (2021). *Giving our characters voices*. Available at: https://blog.duolingo.com/character-voices/ (Accessed July 15, 2024).

[ref21] Christensen K. B. Makransky G. Horton M. (2017). Critical values for Yen’s Q3: identification of local dependence in the Rasch model using residual correlations. Appl. Psychol. Meas. 41, 178–194. doi: 10.1177/0146621616677520, PMID: 29881087 PMC5978551

[ref22] Chukharev-Hudilainen E. Ockey G. J. (2021). The development and evaluation of interactional competence elicitor for oral language assessments. ETS Res. Rep. Ser. 2021, 1–20. doi: 10.1002/ets2.12319

[ref23] Circi R. Hicks J. Sikali E. (2023). Automatic item generation: foundations and machine learning-based approaches for assessments. Front. Educ. 8:858273. doi: 10.3389/feduc.2023.858273

[ref24] Cohen J. (1988). Statistical power analysis for the behavioral sciences. 2nd Edn. Hillsdale, NJ: Lawrence Erlbaum Associates, Publishers.

[ref25] Council of Europe (2020). Common European framework of reference for languages: Learning, teaching, assessment – Companion volume. Strasbourg: Council of Europe Publishing.

[ref26] Dai D. W. (2024). Assessing interactional competence: Principles, test development and validation through an L2 Chinese IC test. Berlin, Germany: Peter Lang.

[ref27] Davis L. (2009). The influence of interlocutor proficiency in a paired oral assessment. Lang. Test. 26, 367–396. doi: 10.1177/0265532209104667

[ref28] Devlin J. Chang M. W. Lee K. Toutanova K. (2019). *BERT: pre-training of deep bidirectional transformers for language understanding*. In: Proceedings of the 2019 Conference of the North American Chapter of the Association for Computational Linguistics: Human Language Technologies, Volume 1 (Long and Short Papers). Minneapolis, Minnesota: Association for Computational Linguistics, 4171–4186.

[ref29] Dinan E. Roller S. Shuster K. Fan A. Auli M. Weston J. (2019). *Wizard of Wikipedia: Knowledge-powered conversational agents*. In: International Conference on Learning Representations.

[ref30] Dombi J. Sydorenko T. Timpe-Laughlin V. (2022). Common ground, cooperation, and recipient design in human-computer interactions. J. Pragmat. 193, 4–20. doi: 10.1016/j.pragma.2022.03.001

[ref31] Downing S. M. Haladyna T. M. (2006). Handbook of test development. New York: Routledge.

[ref32] Embretson S. Yang X. (2006). “Automatic item generation and cognitive psychology” in Handbook of statistics (Amsterdam, Netherlands: Elsevier), 747–768.

[ref33] Eskénazi M. (2009). An overview of spoken language technology for education. Speech Comm. 51, 832–844. doi: 10.1016/j.specom.2009.04.005

[ref34] Evanini K. So Y. Tao J. Zapata-Rivera D. Luce C. Battistini L.. (2014). “Performance of a trialogue-based prototype system for English language assessment for young learners” in 4st workshop on Child, computer and interaction, WOCCI 2014. eds. Berkling K. Giuliani D. Potamianos A. (Singapore: ISCA), 79–84.

[ref35] Galaczi E. D. (2008). Peer-peer interaction in a speaking test: the case of the first certificate in English examination. Lang. Assess. Q. 5, 89–119. doi: 10.1080/15434300801934702

[ref36] Galaczi E. Taylor L. (2018). Interactional competence: Conceptualisations, operationalisations, and outstanding questions. Lang. Assess. Q. 15, 219–236. doi: 10.1080/15434303.2018.1453816

[ref37] Gierl M. J. Haladyna T. M. (2012). Automatic item generation: Theory and practice. Abingdon: Routledge.

[ref38] Gokturk N. Chukharev E. (2024). Exploring the potential of a spoken dialog system-delivered paired discussion task for assessing interactional competence. Lang. Assess. Q. 21, 60–99. doi: 10.1080/15434303.2023.2289173

[ref39] Gopalakrishnan K. Hedayatnia B. Chen Q. Gottardi A. Kwatra S. Venkatesh A.. (2019). *Topical-chat: towards knowledge-grounded open-domain conversations*. In Proceeding Interspeech 2019. pp. 1891–1895.

[ref40] Haladyna T. M. (2013). “Automatic item generation: a historical perspective” in Automatic item generation: Theory and practice. eds. Gierl M. J. Haladyna T. M. (New York: Routledge), 13–25.

[ref41] Han J.-E. Koh J.-S. Seo H.-T. Chang D.-S. Sohn K.-A. (2024). *PSYDIAL: personality-based synthetic dialogue generation using large language models*. In: Proceedings of the 2024 joint international conference on computational linguistics, language resources and evaluation (LREC-COLING 2024), pp. 13321–13331.

[ref42] Hartman G. (2020). *Building character: How a cast of characters can help you learn a language*. Available at: https://blog.duolingo.com/building-character/ (Accessed July 15, 2024).

[ref43] Higashinaka R. Funakoshi K. Kobayashi Y. Inaba M. (2016). *The dialogue breakdown detection challenge: task description, datasets, and evaluation metrics*. In: Proceedings of the Tenth International Conference on Language Resources and Evaluation (LREC’16), pp. 3146–3150.

[ref44] Hou Y. Fang M. Che W. Liu T. (2019). *A corpus-free State2Seq user simulator for task-oriented dialogue*. In: Chinese computational linguistics: 18th China National Conference, CCL 2019, Kunming, China, October 18–20, 2019, proceedings. Berlin, Heidelberg: Springer-Verlag, pp. 689–702.

[ref45] House J. Kádár D. Z. (2023). Speech acts and interaction in second language pragmatics: a position paper. Lang. Teach. 1–12, 1–12. doi: 10.1017/S0261444822000477

[ref46] Irvine S. H. Kyllonen P. C. (2002). Item generation for test development, vol. 10. Hillsdale, NJ: Lawrence Erlbaum Associates Publishers, 487–491.

[ref47] Iwashita N. (1996). *The validity of the paired interview format in oral performance assessment*. Melbourne Papers in Language Testing, No. 5. pp. 51–66.

[ref48] Ji Z. Lee N. Frieske R. Yu T. Su D. Xu Y.. (2023). Survey of hallucination in natural language generation. ACM Comput. Surv. 55, 1–38. doi: 10.1145/3571730

[ref49] Jiang A. Q. Sablayrolles A. Mensch A. Bamford C. Chaplot D. S. De Las Casas D.. (2023). Mistral 7B. Available at: http://arxiv.org/abs/2310.06825 (Accessed July 17, 2024).

[ref50] Jiang G. Xu M. Zhu S.-C. Han W. Zhang C. Zhu Y. (2024). Evaluating and inducing personality in pre-trained language models. Proceedings of the 37th international conference on neural information processing systems. Red Hook, NY: Curran Associates Inc.

[ref51] Johnson M. Tyler A. (1998). “Re-analyzing the OPI: how much does it look like natural conversation?” in Studies in bilingualism. eds. Young R. He A. W. (Amsterdam: John Benjamins Publishing Company), 27.

[ref52] Jucker A. H. (2017). Speech acts and speech act sequences: greetings and farewells in the history of American English. Stud. Neophilol. 89, 39–58. doi: 10.1080/00393274.2017.1358662

[ref53] Karatay Y. (2022). Development and validation of spoken dialog system-based oral communication tasks in an ESP context. Ames, IA: Iowa State University.

[ref54] Kim H. Hessel J. Jiang L. West P. Lu X. Yu Y.. (2023). SODA: million-scale dialogue distillation with social commonsense contextualization. In: Bouamor H. Pino J. Bali K. Proceedings of the 2023 Conference on Empirical Methods in Natural Language Processing. Singapore: Association for Computational Linguistics, pp. 12930–12949.

[ref55] Kormos J. (1999). Simulating conversations in oral-proficiency assessment: a conversation analysis of role plays and non-scripted interviews in language exams. Lang. Test. 16, 163–188. doi: 10.1177/026553229901600203

[ref56] Kosh A. E. Simpson M. A. Bickel L. Kellogg M. Sanford-Moore E. (2018). A cost–benefit analysis of automatic item generation. Educ. Measur. 38, 48–53. doi: 10.1111/emip.12237

[ref57] Kumar A. Agarwal C. Srinivas S. Li A. J. Feizi S. Lakkaraju H. (2024). *Certifying LLM safety against adversarial prompting*. Available at: http://arxiv.org/abs/2309.02705 (Accessed July 17, 2024).

[ref58] Lee Y.-J. Lim C.-G. Choi Y. Lm J.-H. Choi H.-J. (2022). “PERSONACHATGEN: generating personalized dialogues using GPT-3” in Proceedings of the 1st workshop on customized chat grounding persona and knowledge. eds. Lim H. Kim S. Lee Y. Lin S. Seo P. H. Suh Y. (Gyeongju, Republic of Korea: Association for Computational Linguistics), 29–48.

[ref59] Li M. Roller S. Kulikov I. Welleck S. Boureau Y.-L. Cho K.. (2020). “Don’t say that! Making inconsistent dialogue unlikely with unlikelihood training” in Proceedings of the 58th annual meeting of the Association for Computational Linguistics. eds. Jurafsky D. Chai J. Schluter N. Tetreault J. (United States: Association for Computational Linguistics), 4715–4728.

[ref60] Litman D. Lim G. S. Strik H. (2018). Speech technologies and the assessment of second language speaking: approaches, challenges, and opportunities. Lang. Assess. Q. 15, 294–309. doi: 10.1080/15434303.2018.1472265

[ref61] Liu S. Zheng C. Demasi O. Sabour S. Li Y. Yu Z.. (2021). “Towards emotional support dialog systems” in Proceedings of the 59th Annual Meeting of the Association for Computational Linguistics and the 11th International Joint Conference on Natural Language Processing. eds. Zong C. Xia F. Li W. Navigli R., vol. 1 (Association for Computational Linguistics), 3469–3483.

[ref62] Lynch C. J. Jensen E. J. Zamponi V. O’Brien K. Frydenlund E. Gore R. (2023). A structured narrative prompt for prompting narratives from large language models: sentiment assessment of chatgpt-generated narratives and real tweets. Future Internet 15:375. doi: 10.3390/fi15120375

[ref63] Lyu K. Zhao H. Gu X. Yu D. Goyal A. Arora S. (2024). *Keeping LLMs aligned after fine-tuning: The crucial role of prompt templates*. Available at: http://arxiv.org/abs/2402.18540 (Accessed July 22, 2024).

[ref64] Mohapatra B. Pandey G. Contractor D. Joshi S. (2021). “Simulated chats for building dialog systems: learning to generate conversations from instructions” in Findings of the Association for Computational Linguistics: EMNLP 2021, virtual event/Punta Cana, Dominican Republic, 16–20 November, 2021. eds. Moens M.-F. Huang X. Specia L. Yih S. W. (Pennsylvania: Association for Computational Linguistics), 1190–1203.

[ref65] Nakatsuhara F. (2011). Effects of test-taker characteristics and the number of participants in group oral tests. Lang. Test. 28, 483–508. doi: 10.1177/0265532211398110

[ref66] Ockey G. J. Chukharev-Hudilainen E. (2021). Human versus computer partner in the paired oral discussion test. Appl. Linguis. 42, 924–944. doi: 10.1093/applin/amaa067

[ref67] Ockey G. J. Chukharev-Hudilainen E. Hirch R. R. (2023). Assessing interactional competence: ICE versus a human partner. Lang. Assess. Q. 20, 377–398. doi: 10.1080/15434303.2023.2237486

[ref1] OpenAI Achiam J. Adler S. Agarwal S. Ahmad L. Akkaya I.. (2024). *GPT-4 Technical Report*. Available at: http://arxiv.org/abs/2303.08774 (Accessed July 15, 2024).

[ref68] Papageorgiou S. Davis L. Norris J. M. Gomez P. G. Manna V. F. Monfils L. (2021). *Design framework for the TOEFL® essentials™ test*.

[ref69] Papangelis A. Wang Y.-C. Molino P. Tur G. (2019). “Collaborative multi-agent dialogue model training via reinforcement learning” in Proceedings of the 20th annual SIGdial meeting on discourse and dialogue. eds. Nakamura S. Gasic M. Zukerman I. Skantze G. Nakano M. Papangelis A. (Stockholm, Sweden: Association for Computational Linguistics), 92–102.

[ref70] Park Y. Lee S. Shin S.-Y. (2022). Developing a local academic English listening test using authentic unscripted audio-visual texts. Lang. Test. 39, 401–424. doi: 10.1177/02655322221076024

[ref71] Plough I. Banerjee J. Iwashita N. (2018). Interactional competence: genie out of the bottle. Lang. Test. 35, 427–445. doi: 10.1177/0265532218772325

[ref72] Radford A. Wu J. Child R. Luan D. Amodei D. Sutskever I.. (2019). *Language models are unsupervised multitask learners*. OpenAI blog, pp. 1–9.

[ref73] Ramanarayanan V. Suendermann-Oeft D. Lange P. Ivanov A. V. Evanini K. Yu Z.. (2016). Bootstrapping development of a cloud-based spoken dialog system in the educational domain from scratch using crowdsourced data. ETS Res. Rep. Ser. 2016, 1–7. doi: 10.1002/ets2.12105

[ref74] Rashkin H. Reitter D. Tomar G. S. Das D. (2021). “Increasing faithfulness in knowledge-grounded dialogue with controllable features” in Proceedings of the 59th Annual Meeting of the Association for Computational Linguistics and the 11th International Joint Conference on Natural Language Processing, ACL/IJCNLP 2021. eds. Zong C. Xia F. Li W. Navigli R. (Stockholm, Sweden: Association for Computational Linguistics), 704–718.

[ref75] Reimers N. Gurevych I. (2019). “Sentence-BERT: sentence Embeddings using Siamese BERT-networks” in Proceedings of the 2019 Conference on Empirical Methods in Natural Language Processing and the 9th International Joint Conference on Natural Language Processing (EMNLP-IJCNLP). eds. Inui K. Jiang J. Ng V. Wan X. (Hong Kong, China: Association for Computational Linguistics), 3982–3992.

[ref76] Roever C. Dai D. W. (2021). “Reconceptualising interactional competence for language testing” in Assessing speaking in context. eds. Salaberry M. R. Burch A. R. (Bristol, UK: Multilingual Matters), 23–49.

[ref77] Roever C. Kasper G. (2018). Speaking in turns and sequences: interactional competence as a target construct in testing speaking. Lang. Test. 35, 331–355. doi: 10.1177/0265532218758128

[ref78] Saville N. McElwee S. (2021). “Quality management in test production and administration” in The Routledge handbook of language testing. eds. Fulcher G. Harding L. (Abingdon: Routledge), 597–621.

[ref79] Sayin A. Gierl M. (2024). Using OpenAI GPT to generate reading comprehension items. Educ. Measur. 43, 5–18. doi: 10.1111/emip.12590

[ref80] Seedhouse P. (2013). “Oral proficiency interviews as varieties of interaction” in Assessing second language pragmatics. eds. Ross S. J. Kasper G. (London: Palgrave Macmillan UK), 199–219.

[ref81] Shuster K. Poff S. Chen M. Kiela D. Weston J. (2021). “Retrieval augmentation reduces hallucination in conversation” in Findings of the Association for Computational Linguistics: EMNLP 2021, virtual event / Punta Cana, Dominican Republic, 16–20 November, 2021. eds. Moens M.-F. Huang X. Specia L. Yih S. W. (Hong Kong, China: Association for Computational Linguistics), 3784–3803.

[ref82] Sireci S. G. Zenisky A. L. (2006). “Innovative item formats in computer-based testing: in pursuit of improved construct representation” in Handbook of test development. eds. Downing S. M. Haladyna T. M. (Mahwah, NJ: Erlbaum), 329–347.

[ref83] Song H. Zhang W.-N. Hu J. Liu T. (2020). “Generating persona consistent dialogues by exploiting natural language inference” in The thirty-fourth AAAI conference on artificial intelligence, AAAI 2020, the thirty-second innovative applications of artificial intelligence conference, IAAI 2020, the tenth AAAI symposium on educational advances in artificial intelligence, EAAI 2020. ed. Song H. (New York, USA: AAAI Press), 8878–8885.

[ref84] Staples S. LaFlair G. T. Egbert J. (2017). Comparing language use in oral proficiency interviews to target domains: conversational, academic, and professional discourse. Mod. Lang. J. 101, 194–213. doi: 10.1111/modl.12385

[ref85] Su P. Wu C.-H. Lee L.-S. (2015). *A recursive dialogue game for personalized computer-aided pronunciation training*. In: IEEE/ACM Transactions on Audio, Speech, and Language Processing, vol. 23, 1–141.

[ref86] Suárez-Álvarez J. Oliveri M. E. Sireci S. G. (2023). *DIRTy CATs and other DIRTy assessments: the adult skills assessment program*. In: Proceedings of the annual meeting of the National Council on measurement in education, Chicago, IL.

[ref87] Sun B. Li Y. Mi F. Bie F. Li Y. Li K. (2023). “Towards fewer hallucinations in knowledge-grounded dialogue generation via augmentative and contrastive knowledge-dialogue” in Proceedings of the 61st annual meeting of the Association for Computational Linguistics. eds. Rogers A. Boyd-Graber J. Okazaki N., vol. 2 (Toronto, Canada: Association for Computational Linguistics), 1741–1750.

[ref88] Suvorov R. Li Z. (2023). *Investigating effect of interactive videos on test-takers’ performance. British Council, Cambridge assessment English and IDP*: IELTS Australia. Available at: https://ielts.org/researchers/our-research/research-reports/investigating-the-effect-of-interactive-videos-on-test-takers-performance-on-the-listening-section-of-ielts (Accessed July 29, 2024).

[ref89] Timpe-Laughlin V. Evanini K. Green A. Blood I. Dombi J. Ramanaranayan V. (2017). *Designing interactive, automated dialogues for L2 pragmatics learning*. SEMDIAL 2017 SaarDial, No. 143.

[ref90] Timpe-Laughlin V. Sydorenko T. Daurio P. (2020). Using spoken dialogue technology for L2 speaking practice: what do teachers think? Comput. Assist. Lang. Learn. 35, 1194–1217. doi: 10.1080/09588221.2020.1774904

[ref91] Touvron H. Lavril T. Izacard G. Martinet X. Lachaux M.-A. Lacroix T.. (2023). *LLaMA: Open and efficient foundation language models*. Available at: http://arxiv.org/abs/2302.13971 (Accessed July 17, 2024).

[ref92] van der Linden W. J. Glas C. A. (2010). Elements of adaptive testing. New York: Springer.

[ref93] van Lier L. (1989). Reeling, writhing, drawling, stretching, and fainting in coils: Oral proficiency interviews as conversation. TESOL Q. 23:489. doi: 10.2307/3586922

[ref94] Vaswani A. Shazeer N. Parmar N. Uszkoreit J. Jones L. Gomez A. N.. (2017). *Attention is all you need*. In: Advances in neural information processing systems.

[ref95] von Davier M. (2018). Automated item generation with recurrent neural networks. Psychometrika 83, 847–857. doi: 10.1007/s11336-018-9608-y, PMID: 29532403

[ref96] Wagner E. (2014). “Assessing Listening” in The companion to language assessment. ed. Wagner E. (New York: John Wiley & Sons, Ltd.), 47–63.

[ref97] Wei A. Haghtalab N. Steinhardt J. (2023). “Jailbroken: how does LLM safety training fail?” in Advances in neural information processing systems. eds. Oh A. Naumann T. Globerson A. Saenko K. Hardt M. Levine S. (New York: Curran Associates, Inc.), 80079–80110.

[ref98] Whitely S. E. (1983). Construct validity: construct representation versus nomothetic span. Psychol. Bull. 93, 179–197. doi: 10.1037/0033-2909.93.1.179

[ref99] Wu Q. Feng S. Chen D. Joshi S. Lastras L. Yu Z. (2022). “DG2: data augmentation through document grounded dialogue generation” in Proceedings of the 23rd annual meeting of the special interest group on discourse and dialogue. eds. Lemon O. Hakkani-Tur D. Li J. J. Ashrafzadeh A. Garcia D. H. Alikhani M. (Edinburgh, UK: Association for Computational Linguistics), 204–216.

[ref100] Xie Y. Yi J. Shao J. Curl J. Lyu L. Chen Q.. (2023). Defending ChatGPT against jailbreak attack via self-reminders. Nat Mach Intell 5, 1486–1496. doi: 10.1038/s42256-023-00765-8

[ref101] Xu J. Ung M. Komeili M. Arora K. Boureau Y.-L. Weston J. (2023). “Learning new skills after deployment: improving open-domain internet-driven dialogue with human feedback” in Proceedings of the 61st annual meeting of the Association for Computational Linguistics. eds. Rogers A. Boyd-Graber J. L. Okazaki N. (Toronto, Canada: Association for Computational Linguistics), 13557–13572.

[ref102] Yen W. M. (1984). Effects of local item dependence on the fit and equating performance of the three-parameter logistic model. Appl. Psychol. Meas. 8, 125–145. doi: 10.1177/014662168400800201

[ref103] Yen W. M. (1993). Scaling performance assessments: strategies for managing local item dependence. J. Educ. Meas. 30, 187–213. doi: 10.1111/j.1745-3984.1993.tb00423.x

[ref104] Youn S. J. (2020). Managing proposal sequences in role-play assessment: validity evidence of interactional competence across levels. Lang. Test. 37, 76–106. doi: 10.1177/0265532219860077

[ref105] Young R. He A. W. (1998). Talking and testing: Discourse approaches to the assessment of Oral proficiency. Amsterdam, Netherlands: John Benjamins Publishing.

[ref106] Zhang S. Dinan E. Urbanek J. Szlam A. Kiela D. Weston J. (2018). “Personalizing dialogue agents: I have a dog, do you have pets too?” in Proceedings of the 56th annual meeting of the Association for Computational Linguistics. eds. Gurevych I. Miyao Y., vol. 1 (Melbourne, Australia: Association for Computational Linguistics), 2204–2213.

[ref107] Zheng C. Sabour S. Wen J. Zhang Z. Huang M. (2023). “AugESC: dialogue augmentation with large language models for emotional support conversation” in Findings of the Association for Computational Linguistics: ACL 2023, July 9–14, 2023. eds. Rogers A. Boyd-Graber J. L. Okazaki N. (Toronto, Canada: Association for Computational Linguistics), 1552–1568.

[ref108] Zu J. Choi I. Hao J. (2023). Automated distractor generation for fill-in-the-blank items using a prompt-based learning approach. Psychol. Test. Assess. Model. 65, 55–75.

