# Supplementary material for: A generative AI-driven interactive listening assessment task
Source: Front Artif Intell. 2024 Nov 4;7:1474019. doi: 10.3389/frai.2024.1474019 (PMC11571064; doi:10.3389/frai.2024.1474019)
Supplement: Supplementary file 1 [file Data_Sheet_1.docx]

**Appendix**

**Appendix A: Scenario Expansion Prompts**

**Student-Student**

Rewrite the scenario description between two students to be in second person. Where appropriate, include details about the class or course of study the students are involved in. Also include personal details about each of the students involved that might provide more context about the conversation.

Here's an example:

Original: One student is writing a paper and asking for feedback on it from their friend.

Revised: Your friend is writing a paper for their introductory psychology class and is looking for some feedback on it. They ask you to take a look at it, since you took the class last semester.

===

Original: Two students are discussing an upcoming test and how they plan to study for it.

Revised: You and your classmate are talking about how you plan to study for an upcoming test in your Anthropology class. You've both been doing alright in the class, but want to put in a lot of effort to get better grades on this test.

===

Original: Two first-year students are discussing where they might want to study abroad in a couple of years.

Revised: You and your first-year friend are discussing where you might want to study abroad in a couple of years. You both want to go somewhere in Europe, but are trying to decide between a few different countries.

===

Original: A student has been offered the chance to be a teaching assistant, but is unsure about whether they should do it or not.

Revised: You have been offered the chance to be a teaching assistant for your professor's Introduction to Linguistics class next semester, but you are unsure about whether you should do it or not. You would be helping to lead a discussion section and grade assignments for the class.

===

Original: Two students are going over potential majors and minors they could declare.

Revised: You and your friend are meeting up to discuss potential majors and minors you could declare. You both want to major in Biology, but you're also considering a minor in Chemistry. Your friend is thinking about minoring in Mathematics.

**Student-Professor**

Rewrite the scenario description between a student and a professor to be in second person where the student is the reader. Add details describing the class, type of job, course of study, or type of assignment that the scenario describes. Also include information such as the relationship between the student and the professor or personal details about the student that might provide more context about the scenario.

Here's an example:

Original: A student is trying to decide if they should apply to graduate school programs and asks their advisor for advice.

Revised: You are a student in the Biology department at your university and you are trying to decide if you should apply to graduate school programs. You decide to ask your advisor, who you have taken several classes with and who you have often gone to for advice in the past.

===

Original: A student was watching a recorded lecture, but there were problems in the recording so they wanted to know if the professor could go over the material with them.

Revised: You were watching a recording of a lecture for your Archeology class, but there were technical issues and there was no sound for part of the recording. Your professor was describing the history of a certain type of architecture which will be on the midterm.

===

Original: A student is applying for jobs and wants their advisor to look at their resume.

Revised: You are an Engineering student who is about to graduate and you are in the process of applying for jobs. You would like your advisor, who is well-regarded in the field and with whom you have a good relationship, to look over your resume and give you some feedback.

===

Original: A student is missing a lot of class because of their job and wonders if there is any way to still pass the course.

Revised: You are a student in an Introduction to Sociology class. You have been missing a lot of class because you have been working more hours at your job than you originally anticipated. You are starting to fall behind in the coursework and you want to talk with the professor about ways you can still pass the class.

===

Original: A student is interested in doing an independent study with a professor and wants to know what the requirements are.

Revised: You are a junior in college and you are interested in doing an independent study next semester with a professor who specializes in American History. You go to talk with the professor about the requirements for an independent study and what you would need to do to get credit for the class.

**Appendix B: Scenario Subject Identification**

Identify the class, academic task, focus or sub-topic of the task, and/or university program listed in the described scenario. The possible tasks are "Paper", "Exam", "Research", "Project", "Reading", "Thesis".

For example:

===

Scenario: You are a student in Professor Smith's American History class. The next assignment is to write a paper on the American Revolution, and you have a question about the details of the assignment.

Class:American History||Task:Paper||Focus:American Revolution||Program:None

===

Scenario: You are a sophomore who is trying to decide which of two classes to take next semester. One class is about Medieval History and the other is about the History of the Renaissance. You are unsure which one will be more useful for your major and you go to talk with your advisor about it.

Class: Medieval History, History of the Renaissance||Task:None||Focus:None||Program:None

===

Scenario: You are an Education major and you are trying to decide which of two classes to take next semester in order to fulfill your requirements for the major. You go to talk with your advisor about which class would be more useful for you to take.

Class:None||Task:None||Focus:None||Education

===

Scenario: You are a senior English major who would like to do a thesis in your final year. You approach your professor, who is well-known in the department for their work on feminist theory, and ask if they would be willing to advise you.

Class:None||Task:Thesis||Focus:Feminist Theory||Program:English

===

Scenario: You are a student in your professor's Introduction to Psychology class. You were out sick for a week and fell behind on the readings. You were assigned to write a summary of the readings which is due tomorrow, but you feel like you need more time to complete it.

Class:Introduction to Psychology||Task:Reading, Summary||Focus:Psychology||Program:None

===

Scenario: You are a student at your university and you are planning to study abroad in Japan next semester. You have been talking to some other students who have studied abroad and they have told you that it is a really great experience. However, you are worried about how it will affect when you are able to graduate. You decide to ask your advisor about it during their office hours.

Class:None||Task:Study Abroad||Focus:Japan||Program:None

===

Scenario:

**Appendix C: Scenario Rewrite Prompt**

Rewrite the following description, changing the class or academic program to a different, specified subject. Change any details to also make sense based on the new specified subject, including rewriting the name of the class.

Here's an example:

Subject: Biology

Original: You are a student in an American History class. The next assignment is to write a paper on the American Revolution, and you have a question about the details of the assignment.

Rewritten: You are a student in a Microbiology class. The next assignment is to write a paper on gut bacteria, and you have a question about the details of the assignment.

Now you try:

Subject: Metalworking

Original: You are a student in Professor Smith's American History class. The professor has just announced that the next assignment will be a research paper on any topic related to the Civil War. You raise your hand and when called on, you ask the professor for clarification about what kinds of sources will be acceptable for the assignment.

Rewritten: You are a student in Professor Smith's Welding and Smelting class. The professor has just announced that the next assignment will be a research paper on any topic related to the different types of metal alloys. You raise your hand and when called on, you ask the professor for clarification about what kinds of sources will be acceptable for the assignment.

===

Subject: Computer Science

Original: You are a sophomore who is trying to decide which of two classes to take next semester. One class is about Medieval History and the other is about the History of the Renaissance. You are unsure which one will be more useful for your major and you go to talk with your advisor about it.

Revised: You are a sophomore who is trying to decide which of two classes to take next semester. One class is about Data Structures and the other is about Algorithms. You are unsure which one will be more useful for your major and you go to talk with your advisor about it.

===

Subject: American History

Original: You are a student in Professor Taylor's English class. The class is small, with only fifteen students, and you have gotten to know the professor well over the course of the semester. You have a question about the details of an upcoming assignment and decide to go to office hours to talk to the professor about it.

Revised: You are a student in Professor Taylor's Early American Colonial History class. The class is small, with only fifteen students, and you have gotten to know the professor well over the course of the semester. You have a question about the details of an upcoming assignment and decide to go to office hours to talk to the professor about it.

===

Subject:

**Appendix D: Conversation Generation**

**Student-Student**

Write a conversation between you and another student at your university, based on a given scenario description. The scenario will describe the goal that the two of you are trying to accomplish in the conversation, as well as details about specific classes, subjects and other personal details that provide additional context and that you should include in the conversation. The conversation should have least 9 lines of dialogue.

===

Scenario: You and your classmate are discussing the upcoming test in your Ancient Civilizations class. You're both a little worried about it, since you didn't do as well as you wanted to on the last test. You decide to study together for this one.

***

Classmate: Hey, how's the studying going for the Ancient Civilizations test next week?

You: Not great. I can't keep track of all the different ages and emperors, it's way too much.

Classmate: Yeah, I know what you mean. There are so many people and dates and battles that I'm having trouble keeping them all straight.

You: At this rate I'll be lucky to pass, let alone get a good grade.

Classmate: I've been trying to put together a study guide to help me, do you want to try to go over it together?

You: Sure, I've been doing the same so maybe we can put them together and make sure we've got everything covered.

Classmate: Great, when do you want to meet up?

You: Let's meet in the library tomorrow afternoon and see if we can at least get up through the start of the Roman Empire.

Classmate: That sounds like a great idea. I'll see you tomorrow then.

You: Oh wait, sorry, I just remembered I agreed to meet with another friend tomorrow afternoon. Can you meet before lunch tomorrow?

Classmate: Yeah, that works.

You: Great, thanks, see you then!

===

Scenario: You are considering changing your major from English to Creative Writing, and are looking for advice from your friend who is already a Creative Writing major. They tell you about the workload for the major and their experience with the department so far.

***

You: Hey, I've been thinking about changing my major to Creative Writing. What do you think?

Classmate: It's a lot of work, but I love it. I really can't recommend it enough.

You: That's good to hear. I've been thinking about it for a while, but I don't know if I'm up for the workload.

Classmate: My first year was really rough, I think I was writing a couple thousand words a week across all of my classes. And there's a lot of reading on top of that.

You: Hmm, I don't know if I'm ready for that. I like writing, but I don't know if I could keep up that pace.

Classmate: It's definitely hard at first, but I love it and I think you would too. Plus, the department is really great. All the professors are really passionate and they really care about their students.

You: Yeah, that's true. I've had some great English professors too. But I don't know, I'm just not sure if I'm ready for that much work.

Classmate: I think you should give it a try. I think you'll really like it.

You: Yeah, maybe you're right. I'll talk to my advisor and see what they think.

Classmate: Let me know what they say! Good luck!

===

Scenario: You and your friend are discussing your work as research assistants for your professors. You are both working on research projects in the Psychology department. You are helping to design experiments and collect data for your professor's project on memory, while your friend is working on a project about bilingualism and language acquisition with their professor.

***

Classmate: Hey, been a while. How's your researching going?

You: Oh, not too bad. I think I told you that I'm working with Professor Johnson, right?

Classmate: Yeah, I think you told me last time we talked.

You: We're working on designing an experiment to study how people remember different types of information.

Classmate: That sounds really interesting. I'm working on a project with Professor Nguyen about bilingualism and language acquisition. We're collecting data from bilingual students at the university to see how they learn and use language in different situations.

You: Oh wow, that sounds really cool. I wish my project was that interesting.

Classmate: Your project is interesting! Memory is really important, we wouldn't be able to function without it.

You: Yeah, I guess you're right. It's just a lot of work designing experiments and then running them and trying to collect all the data.

Classmate: Yeah, data collection can be really time-consuming and frustrating, especially when you're trying to get people to participate in your experiments. But it's all worth it in the end when you see the results of your hard work.

You: Yeah, I'm looking forward to seeing the results of our experiments too. Hopefully we'll learn something new about how people remember things.

Classmate: I'm sure you will! Good luck with the rest of your research!

===

**Student-Professor**

Write a conversation between you and a professor at your university, based on a given scenario description. The professor could be your professor for a class, your advisor, or any other professor at the university. The scenario will describe why you are talking to the professor, as well as details about specific classes, subjects and other personal details that provide additional context and that you should include in the conversation. The professor's lines should have one or two substantially longer lines of dialogue, and the conversation should have least 9 lines of dialogue total.

===

Scenario: You are a student in the Political Science department and you want to do a thesis in your final year. You approach your professor, who is an expert in the field of international relations, and ask if they would be willing to advise you.

***

Professor: Hi there! What can I do for you?

You: Hi, Professor. I'm a Political Science student and I'm interested in doing a thesis in my final year. I was wondering if you would be willing to advise me.

Professor: Potentially, can you tell me a little bit about what area of research you're interested in?

You: I'm interested in the role of the UN in peacekeeping operations.

Professor: That's a good area with a wide range of potential topics. I think you would need to take a few classes before you're ready to tackle a thesis on that though. Have you taken any courses on international organizations or international law?

You: Yes, I've taken both of those courses.

Professor: Okay, that's good. If you haven't already, you should also take a look at some of the research that's been done on the topic already and see what other scholars have said about it. Once you have a better understanding of the existing literature, we can start talking about your thesis proposal.

You: Okay, I have a couple ideas based on my past courses and some background research I've done. Would it help if I wrote them up and we discussed them?

Professor: Yes, that would be helpful. I can give you some feedback on your ideas and help you develop them further. Let's set up a meeting next week to talk about your proposal.

===

Scenario: You are a nursing student and you have an assignment due tomorrow that you have not started yet. You go to your professor and explain the situation, asking if you can have an extension on the assignment.

***

You: Professor, I'm sorry to bother you but I'm a bit desperate. I wanted to ask you about the assignment due tomorrow.

Professor: The one on caring for elderly patients?

You: Yes, I haven't been able to start it yet because I've had some long shifts at my internship at the hospital. Is there any way I can have an extension on it?

Professor: Let me see... the assignment is worth a significant portion of your grade for the course, so I can't just give you an extension without a good reason.

You: I understand. It's just that I've been really busy and I haven't had time to work on it.

Professor: Have you tried working on it during your break times at the hospital?

You: Yeah, but those are usually pretty short and I'm usually pretty tired by the end of my shift.

Professor: Well, you could try working on it in the morning before your shifts start.

You: That's not really possible either because I have a long commute and my shifts start early in the morning.

Professor: Okay, let me think about it for a bit. In light of your circumstances, I'll give you an extension until next Monday. But please try to get started on it as soon as possible so that you're not too behind.

You: Thank you so much professor! I really appreciate it and I won't let this happen again!

===

Scenario: You are a student who is about to graduate with a degree in computer science. You have been searching for jobs for a few months but you haven't had any luck. You decide to ask your professor if they have any suggestions or advice on how to go about searching for a job in the computer science field.

***

You: Professor, do you have a minute? I wanted to ask for your advice about something.

Professor: Of course, what is it?

You: I've been searching for a job as a software engineer for a few months but I haven't had any luck. I was wondering if you had any suggestions for finding a job or knew of any openings through your own connections.

Professor: Well, the computer science field is very competitive so you need to make sure that your resume and cover letter are top notch. Have you tried networking with other computer science professionals?

You: Yeah, I've gone to some meetups and events but I haven't really connected with anyone.

Professor: That's not surprising. It can be tough to network when you don't know anyone in the field yet. I know the department has been forwarding job postings that our alumni send us. Have you been checking those out?

You: Yeah, I've applied to a few but I haven't heard back from any of them.

Professor: Have you tried reaching out to the companies after you've applied?

You: No, I didn't think that would be necessary.

Professor: It couldn't hurt. Sometimes it's not just enough to send in a resume through the online portal. Being proactive and reaching out to a recruiter or an alumni who works at the company can sometimes do the trick.

You: I hadn't thought of that. Thank you Professor, I'll give it a try!

===

**Student-Professor, Academic Topic**

Write a dialogue between a student and a professor where a student meets with a professor, either after class or in their office hours, and asks them to explain or describe a person's importance or contributions, a type of plant or animal, or a concept related to a given topic. The professor should give the student a short lecture on the topic, with lines that are significantly longer than the student's. The student should ask two or more clarifying questions about what the professor says.

For example:

===

Topic: "statistics"

***

Student: Hi Professor, thanks for taking the time to see me during your office hours.

Professor: Of course, what can I help you with?

Student: I wanted to ask you about the concept you were discussing in lecture yesterday. I'm struggling to wrap my head around it.

Professor: Sure, which concept was that?

Student: You were talking about the difference between a correlation and a causation.

Professor: Ah, yes. Well, a correlation is when two things seem to be related to each other in some way. For example, you might see that students who study more tend to get better grades, or you might notice that people who live in cold climates tend to drink more hot beverages. A causation is when one thing causes another. So, for example, if you see that people who eat ice cream tend to get brain freeze, you can say that eating ice cream causes brain freeze.

Student: So, could we say that studying more causes better grades?

Professor: Not necessarily. That's where the distinction between correlation and causation comes in. While it's true that there's a relationship between studying and grades, we don't know if one causes the other. It's possible that there's a third factor involved, like being naturally smart, that allows some students to both study more and get better grades.

Student: Oh, I see. So, a correlation just means that two things are related, but causation means that one thing actually causes the other.

Professor: Exactly.

Student: Then how can you prove that one thing causes another?

Professor: Well, usually you would want to do an experiment. For example, you could take a group of students and have half of them study more while the other half studies the same amount as usual. If the group that studied more gets better grades, then you would have some evidence that studying causes better grades.

Student: That makes sense. Thank you for taking the time to explain that to me.

===

Topic: "french philosophers"

***

Professor: Hello there, come on in.

Student: Hi Professor, I had a question about something that came up in the readings you assigned.

Professor: Sure, what was it about?

Student: Could we potentially go over the differences between the philosophies of Jean-Jacque Rousseau and Voltaire? I'm not sure I fully understood them from the textbook.

Professor: Sure, I'd be happy to. Jean-Jacques Rousseau was a philosopher who believed that humans are naturally good but are corrupted by society. He thought that people should return to nature and live simple lives. Then there's Voltaire, who was a philosopher who believed in reason and tolerance. He thought that people should be free to express their opinions and beliefs without fearing persecution.

Student: So, Rousseau thought that humans were good but Voltaire thought that they were reasonable?

Professor: Yes, exactly. Rousseau believed in the goodness of human nature while Voltaire believed in the power of reason.

Student: And what did they think about society?

Professor: Rousseau thought that society corrupted humans while Voltaire thought that it should be a place where people can freely express themselves.

Student: Which of their works would you recommend I read if I wanted to better understand their philosophies?

Professor: Well, if you're interested in Rousseau, I would recommend reading his book "Emile, or On Education". If you're interested in Voltaire, I would recommend reading his book "Candide".

Student: Okay great. Thank you so much Professor, this has been really helpful!
